# The additive effect of periodontitis with hypertension on risk of systemic disease and mortality

**DOI:** 10.1002/JPER.21-0621

**Published:** 2022-05-27

**Authors:** Harriet Larvin, Jing Kang, Vishal R. Aggarwal, Sue Pavitt, Jianhua Wu

**Affiliations:** ^1^ School of Dentistry University of Leeds Leeds UK; ^2^ Oral Biology, School of Dentistry University of Leeds Leeds UK; ^3^ Leeds Institute for Data Analytics University of Leeds Leeds UK

**Keywords:** hypertension, mortality, noncommunicable diseases, oral health, periodontitis, risk factors

## Abstract

**Background:**

Recent evidence suggests that periodontitis (PD) causes hypertension, which is a precursor to development of other systemic diseases. The aim of this study was to examine the effect of hypertension and PD on the risk of subsequent systemic disease.

**Methods:**

This longitudinal cohort study included 244,393 UK Biobank participants who were free of systemic disease other than hypertension at baseline. Self‐reported responses of painful gums or loose teeth were surrogates for PD. Hypertensives were identified by clinical diagnosis, or elevated blood pressure (≥140/90 mmHg). Systemic diseases including cancer, cardiovascular disease (CVD), and diabetes were identified from linked diagnostic codes. Multivariable Cox proportional hazard models were used to quantify the risk of systemic diseases and all‐cause mortality, stratified by hypertensive and PD status.

**Results:**

The average age of the study population was 55.4 years (standard deviation [SD:] 8.1 years), and 130,220 (53.3%) participants were female. At baseline, 131,566 (53.8%) participants were hypertensive and 4.5% reported PD. The incidence rates of all systemic diseases were higher in hypertensive than non‐hypertensive participants of the same PD status. In hypertensives, an additive effect was observed for PD on the risks of CVD (adjusted hazard ratio [HR]: 1.35, 95% confidence interval [CI]: 1.21–1.53) and respiratory disease (HR: 1.11, 95% CI: 0.95–1.30) compared to hypertensive healthy controls.

**Conclusions:**

Hypertensives with PD have exacerbated risks of several systemic diseases. Future interventional studies should consider the effect of periodontal treatment on systemic outcomes in targeted hypertensive populations.

## 1 INTRODUCTION

Prevalence of periodontitis (PD) is widespread; as a chronic oral condition, it is ranked as the sixth most prevalent disease worldwide.^1^ PD is a multifactorial disease, linked to poor oral hygiene and a number of risk factors including older age, being male, and smoking.^2^, ^3^, ^4^ PD induces systemic inflammation and eventual tooth loss as the condition develops into its most severe form.^5^ There is growing evidence that PD is associated to an increased risk of systemic diseases, including diabetes, cardiovascular disease (CVD), and even adverse coronavirus disease 2019 (COVID‐19) outcomes, which is likely caused by shared inflammatory pathways.^6^, ^7^, ^8^, ^9^ With the advancement of precision/personalized medicine, identification of the individual and combined factors that increase one's susceptibility to systemic disease development is becoming increasingly important.

Systemic disease diagnoses such as CVD, diabetes, and depression frequently occur in the common disease trajectories of PD. A recent study has shown that a hypertension diagnosis is also central to a number of the multimorbid trajectories^10^; while hypertension is also independently associated with multimorbidity.^11^ High levels of inflammatory markers such as C‐reactive protein (CRP) are associated with both hypertension and PD,^12^ and there is evidence to suggest that the link between PD and subsequent hypertension is causal.^13^ As yet, the combined effect of hypertension and PD on the subsequent systemic disease development is unclear.

The UK Biobank is a national resource with over half a million participants; it incorporates demographic and lifestyle variables, baseline oral health status, and linked health outcomes data.^14^ The data from the UK Biobank cohort have enabled study of oral health on several health outcomes including cancer, CVD, diabetes, depression, and COVID‐19.^8^, ^10^, ^15^, ^16^ The burden of hypertension affects up to 50% of the UK Biobank cohort^17^; therefore the UK Biobank provides a demonstratable setting for an epidemiological study comparing outcomes in hypertensive and non‐hypertensive populations. The aim of this study was to examine the effect of hypertension and PD on risk of subsequent systemic diseases.

## 2 MATERIALS AND METHODS

### 2.1 Dataset

This prospective longitudinal study used the UK Biobank dataset, a national resource open to researchers with an approved protocol (ref: 54633). The core UK Biobank dataset contains demographic, biomarker, and lifestyle information on more than 500,000 participants aged between 49 and 60 years old at baseline (2006–2010). Self‐reported oral health status (painful gums, loose teeth, or none of the aforementioned) was measured at baseline via responses to an online questionnaire and is contained in the core UK Biobank dataset. Health outcomes data from hospital episode statistics (HES), general practitioner (GP) and prescription records, as well as the cancer and death registries are linked to the core dataset via a unique participant identifier number. The additional health outcomes data provide information including international classification of diseases—10th edition (ICD‐10) codes, Read (version 2) codes, prescriptions, mortality, and the associated entry dates. Updates of HES, GP, and death data were made available for download until November 2020, September 2019, and December 2020, respectively. All participant information is fully anonymized by UK Biobank before being made available to researchers in the data portal. UK Biobank data collection and research were carried out in accordance with the Declaration of Helsinki. Participants provided informed written consent and may withdraw at any time.^14^

### 2.2 Study population

**FIGURE 1:**
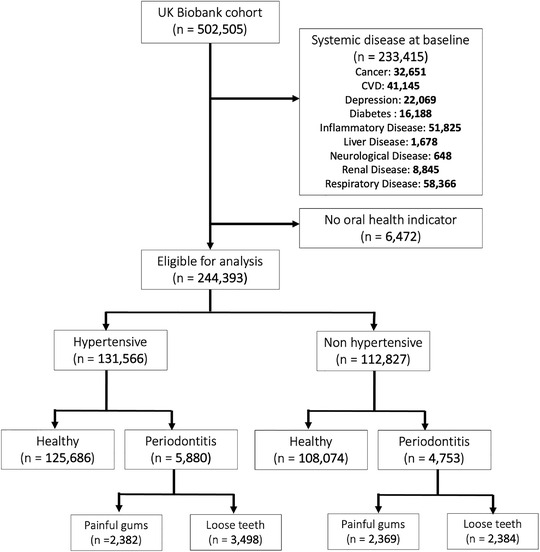
Flow chart showing delivery of exclusion criteria to study population. CVD, cardiovascular disease; PD, periodontitis

All UK Biobank participants with oral health status available and who were free of systemic disease at baseline other than hypertension and PD were included in the study. Systemic diseases included cancers, CVD, depression, diabetes mellitus (diabetes), inflammatory disease, liver disease, neurological disease, renal disease, and respiratory disease and were identified through self‐report at baseline, or by the presence of the ICD‐10 or Read (version 2) codes in health outcomes data prior to study recruitment (for full list see Supplementary Table S1 in the online *Journal of Periodontology*).^18^ Figure 1 demonstrates the study inclusion flow chart.

### 2.3 Study outcomes and exposures

The primary outcome was incident systemic disease diagnosis using codes identified in linked HES or primary care records during follow‐up, and mortality status using civil registration of deaths from Office for National Statistics.

The study exposures were PD and hypertensive status at baseline. Participants were asked “Do you have any of the following?”, where PD was defined by the self‐reported painful gums, or loose teeth, and healthy controls did not report the aforementioned oral health indicators. Painful gums were surrogates of moderate PD while loose teeth suggested more severe PD. Hypertension was confirmed through ICD‐10 or Read (version 2) code identification in health records, or by abnormal blood pressure reading at baseline (above 140/90 mm/Hg). During the baseline visit, two blood pressure readings were measured using automated Omron device 0–255 and mean measurement calculated; where an automated reading was not available, a manual sphyogmometer was used.

### 2.4 Study variables

Baseline information including demographics (age, sex, ethnicity, household income), biomarkers (body mass index (BMI), blood pressure, CRP level), and smoking status was collected at the baseline visit. Self‐reported baseline antihypertensive drug prescriptions were available from interview responses in the core dataset, and prescription codes (Dictionary of Medicines + Devices, Read (version 2) and British National Formulary) were extracted from primary care data (see Supplementary Table S2 in online *Journal of Periodontology*).

### 2.5 Statistical analysis

Baseline characteristics for study participants were summarized using frequency (%) for categorical variables and mean (standard deviation [SD]) for continuous variables, respectively. Subsequent systemic disease during follow‐up was quantified by incidence rate; the number of new cases per 1000 person years, stratified by PD and hypertensive status. Multivariable Cox proportional hazard models were used to quantify the risk of systemic disease development. The models were adjusted for age, sex, BMI, CRP level, household income, history of smoking, time from hypertension diagnosis (years), and antihypertensive prescription. We compared the risk of systemic diseases in non‐hypertensive and hypertensive participants with and without PD (healthy control). We also investigated the risk of systemic disease stratified by PD and hypertension category, respectively. The interaction of hypertension and PD on the overall rate of follow‐up systemic disease was explored in interaction plots.

We assessed the proportional hazard assumptions using Schoenfeld residuals tests. Missing covariate information was handled via multiple imputation by chained equations, and coefficients were combined using Rubin's rule,^19^ using the “mice” package in R.^20^ Sensitivity analyses were conducted to account for the impact of missing data and to assess the effect of antihypertensive prescriptions in hypertensive participants. Significance levels were set to 0.05, and all data processing and analyses were performed in R version 4.0.0.^21^ The study report was written in accordance with STROBE guidelines.

## 3 RESULTS

Overall, there were 244,393 participants included in the final study population; 131,566 (53.8%) participants were hypertensive at baseline and 10,633 (4.4%) participants had self‐reported PD (Figure 1). PD was present 4.5% of hypertensives. The mean age of participants was 55.4 years (SD: 8.1) and 53.3% of participants were female. The average CRP level in the study cohort was 2.20 (SD: 3.71). The mean BMI in the overall study cohort was 26.80 (SD: 4.35) (Table 1).

**TABLE 1:** Summary table of UK Biobank participants stratified by hypertension category and periodontitis indicator

		Non‐hypertensive^a^ *n* = (112,827)			Hypertensive^b^ *n* = (131,566)		
Demographic	Overall (*n* = 244,393)	Healthy (*n* = 108,074)	Painful gums (*n* = 2369)	Loose teeth (*n* = 2384)	Healthy (*n* = 125,686)	Painful gums (*n* = 2382)	Loose teeth (*n* = 3498)
Age, mean (SD)	55.41 (8.13)	52.85 (7.91)	52.51 (7.69)	55.11 (7.84)	57.55 (7.69)	56.97 (7.59)	58.73 (7.05)
Sex, female (%)	130,220 (53.3)	65,249 (60.4)	1587 (67.0)	1254 (52.6)	592,27 (47.1)	1397 (58.6)	1506 (43.1)
Ethnicity, white (%)	230,025 (94.4)	101,718 (94.4)	2040 (86.3)	2026 (85.3)	119,112 (95.1)	2052 (86.4)	3077 (88.4)
Annual household income/£^c^ (%)							
Less than 18,000	37,245 (17.6)	13,381 (14.1)	409 (19.9)	562 (28.1)	37,245 (17.6)	21,513 (20.1)	500 (25.5)
18,000–30,999	51,517 (24.4)	20,489 (21.5)	516 (25.0)	532 (26.6)	51,517 (24.4)	28,520 (26.6)	578 (29.4)
31,000–51,999	58,906 (27.9)	27,435 (28.8)	545 (26.5)	478 (23.9)	58,906 (27.9)	29,282 (27.3)	487 (24.8)
52,000–100,000	49,757 (23.5)	25,876 (27.2)	478 (23.2)	364 (18.2)	49,757 (23.5)	22,307 (20.8)	334 (17.0)
Greater than 100,000	13,884 (6.6)	7962 (8.4)	112 (5.4)	64 (3.2)	13,884 (6.6)	5616 (5.2)	65 (3.3)
CRP/mg/L, mean (SD)	2.20 (3.71)	1.87 (3.45)	2.05 (3.33)	2.44 (3.93)	2.20 (3.71)	2.45 (3.83)	3.01 (4.97)
BMI/kg/m2, mean (SD)	26.80 (4.35)	25.69 (3.91)	25.92 (4.31)	26.27 (4.25)	26.80 (4.35)	27.71 (4.45)	27.81 (4.72)
History of smoking (%)	139,952 (57.3)	60,701 (56.2)	1442 (60.9)	1736 (72.8)	139,952 (57.3)	72,066 (57.3)	1447 (60.7)
Antihypertensive prescription	28,014 (11.5)	11,698 (10.8)	245 (10.3)	240 (10.1)	15,122 (12.0)	309 (13.0)	400 (11.4)

*Note*: Means, medians, and percentages are calculated for variables excluding missing data. There were missing data in the following variables: ethnicity (0.3%), CRP (5.8%), household income (13.5%), and BMI (0.4%).

Abbreviations: BMI, body mass index; CRP, c‐reactive protein; n, number of participants; PD, periodontitis; SD, standard deviation.

^a^ Non‐hypertensive: no history of hypertension and blood pressure within the normal range at baseline.

^b^ Hypertensive: history of hypertension or abnormal blood pressure reading at baseline.

^c^ The exchange rate of GBP to USD at time of writing was £1.00 = $1.36.

### 3.1 Overall risks of systemic diseases

**FIGURE 2:**
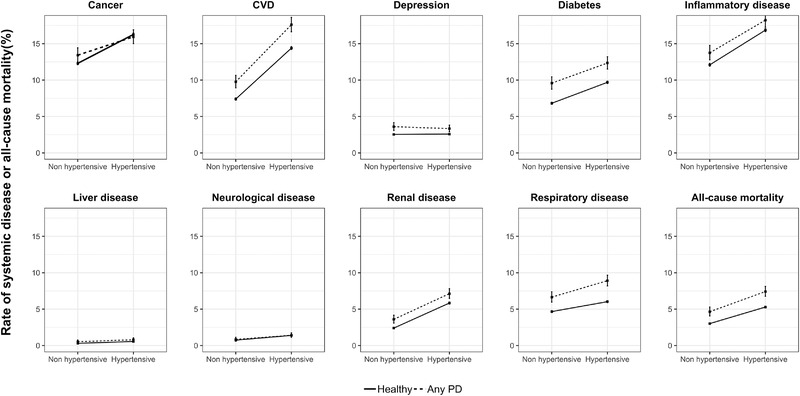
Overall event rate of follow‐up systemic disease or all‐cause mortality by periodontitis status and hypertension category. CVD, cardiovascular disease; PD, periodontitis

The overall median time to the development of any systemic disease was 6.36 years (interquartile range [IQR] = 5.71). Average time to systemic disease development in hypertensive and non‐hypertensive participants was similar (hypertensive: median = 6.45 years, IQR = 5.73; non‐hypertensive: median = 6.18, IQR = 5.66). The rates of all systemic diseases were higher in hypertensive participants than non‐hypertensive, except for depression where the difference was marginal. Interactions between PD and the hypertension category were observed in cancer, depression, and respiratory disease (Figure 2). The overall incidence for neurological disease and liver disease was low (Figure 2 and Table 2). After adjustments, both hypertensive and non‐hypertensive participants with PD had an increased risk of all examined systemic disease outcomes compared to non‐hypertensive healthy controls, except for cancer, inflammatory disease, and neurological disease (Table 3).

**TABLE 2:** Incidence rates of follow‐up systemic disease in study participants per 1000 person years, stratified by hypertension category and periodontitis indicator

		Hypertensive/periodontitis indicator					
		Non‐hypertensive			Hypertensive		
Systemic disease outcome	Cases	Healthy (*n* = 125,686)	Painful gums (*n* = 2382)	Loose teeth (*n* = 3498)	Healthy (*n* = 108,074)	Painful gums (*n* = 2369)	Loose teeth (*n* = 2384)
Cancer	*n*	13291	297	341	20,439	345	592
	IR (95% CI)	10.55 (10.37–10.73)	10.83 (9.63–12.13)	12.42 (11.14–13.82)	14.13 (13.93–14.32)	12.61 (11.32–14.02)	14.89 (13.72–16.14)
CVD	*n*	7995	211	253	18,088	403	632
	IR (95% CI)	6.34 (6.21–6.48)	7.69 (6.69–8.81)	9.22 (8.12–10.43)	12.50 (12.32–12.68)	14.73 (13.33–16.24)	15.90 (14.69–17.19)
Depression	*n*	2742	84	87	3247	94	102
	IR (95% CI)	2.18 (2.10–2.26)	3.06 (2.44–3.79)	3.17 (2.54–3.91)	2.24 (2.17–2.32)	3.44 (2.78–4.21)	2.57 (2.09–3.12)
Diabetes	*n*	7367	239	216	12,182	289	437
	IR (95% CI)	5.85 (5.71–5.98)	8.72 (7.65–9.89)	7.87 (6.86–8.99)	8.42 (8.27–8.57)	10.57 (9.38–11.86)	10.99 (9.99–12.08)
Inflammatory disease	*n*	13,069	317	336	21,189	419	653
	IR (95% CI)	10.37 (10.19–10.55)	11.56 (10.32–12.90)	12.24 (10.97–13.62)	14.64 (14.45–14.84)	15.32 (13.89–16.86)	16.43 (15.19–17.74)
Liver disease	*n*	326	11	14	703	20	27
	IR (95% CI)	0.26 (0.23–0.29)	0.40 (0.20–0.72)	0.51 (0.29–0.86)	0.49 (0.451–0.523)	0.73 (0.447–1.13)	0.68 (0.45–0.99)
Neurological disease	*n*	806	15	25	1746	29	54
	IR (95% CI)	0.64 (0.60–0.69)	0.547 (0.31–0.90)	0.911 (0.59–1.34)	1.21 (1.15–1.26)	1.06 (0.71–1.52)	1.36 (1.02–1.77)
Renal disease	*n*	2588	75	96	7338	161	258
	IR (95% CI)	2.05 (1.98–2.13)	2.73 (2.15–3.43)	3.50 (2.83–4.27)	5.07 (4.96–5.19)	5.89 (5.01–6.87)	6.49 (5.72–7.33)
Respiratory disease	*n*	5043	144	172	7573	197	327
	IR (95% CI)	4.00 (3.89–4.11)	5.25 (4.43–6.18)	6.27 (5.37–7.28)	5.23 (5.12–5.35)	7.20 (6.23–8.28)	8.23 (7.36–9.17)
All‐cause mortality	*n*	3261	88	133	6645	138	298
	IR (95% CI)	2.59 (2.50–2.68)	3.21 (2.57–3.95)	4.85 (4.06–5.74)	4.59 (4.48–4.70)	5.05 (4.24–5.96)	7.50 (6.67–8.40)

*Note*: Incidence rates are out of participants within the same oral health status and hypertension category.

Abbreviations: CI, confidence interval; CVD, cardiovascular disease; IR, incidence per 1000 person years; n, number of cases; PD, periodontitis.

**TABLE 3:** Association between periodontitis and risk of subsequent systemic disease compared to healthy controls, stratified by hypertension category

		Non‐hypertensive			Hypertensive		
Systemic disease/all‐cause mortality	Hazard ratio (95%CI)	Healthy	Painful gums	Loose teeth	Healthy	Painful gums	Loose teeth
Cancer	Crude	1 (ref)	1.03 (0.92–1.15)	1.18 (1.06–1.31)	1.36 (1.33–1.39)	1.20 (1.08–1.33)	1.43 (1.31–1.55)
	Adjusted	1 (ref)	1.04 (0.93–1.17)	1.00 (0.90–1.11)	1.01 (0.98–1.03)	0.92 (0.83–1.03)	0.94 (0.86–1.02)
CVD	Crude	1 (ref)	1.22 (1.07–1.40)	1.46 (1.29–1.66)	2.03 (1.98–2.09)	2.43 (2.20–2.68)	2.60 (2.40–2.82)
	Adjusted	1 (ref)	1.26 (1.10–1.45)^***^	1.17 (1.03–1.33)^*^	1.30 (1.26–1.34)^***^	1.63 (1.47–1.80)^***^	1.43 (1.32–1.55)^***^
Diabetes	Crude	1 (ref)	1.54 (1.36–1.75)	1.36 (1.19–1.55)	1.47 (1.43–1.51)	1.84 (1.64–2.07)	1.91 (1.74–2.11)
	Adjusted	1 (ref)	1.43 (1.26–1.63)^***^	1.12 (0.98–1.29)	1.19 (1.15–1.23)^***^	1.42 (1.26–1.60)	1.34 (1.21–1.48)^***^
Depression	Crude	1 (ref)	1.41 (1.14–1.76)	1.45 (1.17–1.80)	1.03 (0.98–1.08)	1.58 (1.29–1.94)	1.16 (0.95–1.42)
	Adjusted	1 (ref)	1.30 (1.05–1.62)^*^	1.33 (1.07–1.65)^**^	0.97 (0.92–1.03)	1.36 (1.11–1.67)^**^	1.00 (0.82–1.22)
Inflammatory disease	Crude	1 (ref)	1.12 (1.00–1.25)	1.19 (1.06–1.32)	1.44 (1.41–1.47)	1.50 (1.36–1.65)	1.61 (1.49–1.75)
	Adjusted	1 (ref)	1.10 (0.98–1.22)	1.00 (0.89–1.11)	1.00 (0.97–1.02)	1.03 (0.93–1.14)	1.00 (0.92–1.08)
Liver disease	Crude	1 (ref)	1.56 (0.85–2.84)	1.96 (1.15–3.35)	1.88 (1.65–2.14)	2.82 (1.79–4.42)	2.60 (1.76–3.85)
	Adjusted	1 (ref)	1.43 (0.79–2.62)	1.46 (0.85–2.50)	1.26 (1.10–1.46)^**^	1.73 (1.10–2.73)^*^	1.31 (0.88–1.96)
Neurological disease	Crude	1 (ref)	0.86 (0.51–1.43)	1.42 (0.95–2.11)	1.89 (1.74–2.05)	1.65 (1.14–2.39)	2.11 (1.60–2.78)
	Adjusted	1 (ref)	0.85 (0.51–1.42)	0.95 (0.64–1.42)	1.02 (0.93–1.11)	0.95 (0.66–1.38)	0.90 (0.68–1.19)
Renal disease	Crude	1 (ref)	1.34 (1.07–1.69)	1.70 (1.39–2.09)	2.51 (2.40–2.62)	2.91 (2.48–3.41)	3.21 (2.82–3.64)
	Adjusted	1 (ref)	1.30 (1.04–1.64)^*^	1.24 (1.01–1.52)^*^	1.37 (1.31–1.44)^***^	1.53 (1.30–1.79)^***^	1.37 (1.20–1.56)^***^
Respiratory disease	Crude	1 (ref)	1.33 (1.12–1.57)	1.57 (1.35–1.83)	1.31 (1.27–1.36)	1.81 (1.57–2.09)	2.07 (1.85–2.31)
	Adjusted	1 (ref)	1.27 (1.08–1.50)	1.30 (1.12–1.52)^***^	1.02 (0.99–1.07)	1.35 (1.17–1.56)^***^	1.40 (1.25–1.57)^***^
All‐cause mortality	Crude	1 (ref)	1.25 (1.01–1.54)^**^	1.89 (1.59–2.24)	1.79 (1.72–1.87)	1.97 (1.66–2.34)	2.95 (2.62–3.32)
	Adjusted	1 (ref)	0.96 (0.77–1.18)	1.07 (0.90–1.27)	1.01 (0.97–1.06)	1.02 (0.86–1.21)	0.92 (0.81–1.04)

*Note*: Adjusted by age, sex, BMI, ethnicity, average total household income, C‐reactive protein level, history of smoking, time since hypertension diagnosis, and death and hypertension*PD interaction.

Abbreviations: BMI, body mass index; CI, confidence interval; CVD, cardiovascular disease; HR, hazard ratio; PD, periodontitis; ref, reference value.

Statistical significance at the 0.05, 0.01, and 0.001 levels denoted by: *, **, and ***, respectively.

### 3.2 Risk of cancer

**FIGURE 3:**
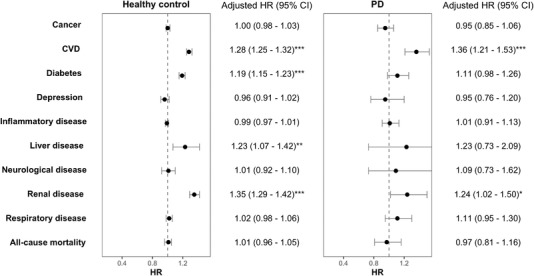
Hazard ratio of systemic disease or all‐cause mortality in hypertensive compared to non‐hypertensive participants, stratified by periodontitis status. CI, confidence interval; CVD, cardiovascular disease; HR, hazard ratio; PD, periodontitis. Adjusted by age, sex, BMI, ethnicity, average total household income, C‐reactive protein level, history of smoking, time since hypertension diagnosis, and antihypertensive prescription. Statistical significance at the 0.05, 0.01, and 0.001 levels denoted by *, **, ***, respectively

An interaction effect between PD and hypertension was observed for risk of cancer (Figure 2). The rate of acquiring cancer during follow‐up was higher in hypertensive healthy controls (incidence rate per 1000 person years [IR]: 14.13, 95% confidence interval [CI]: 13.93–14.32) than in non‐hypertensive healthy controls (IR: 10.55, 95% CI: 10.37–10.73) (Table 2). After adjustments for covariates, the risk of cancer was not changed for hypertensive participants with or without PD (hazard ratio [HR]: 1.00, 95% CI: 0.98–1.03; HR: 0.95, 95% CI: 0.85–1.06) compared to non‐hypertensives (Figure 3).

### 3.3 Risk of CVD

The rate of CVD was approximately halved in non‐hypertensive healthy controls (IR: 6.34, 95% CI: 6.21–6.48) than in hypertensive healthy controls (IR: 12.50, 95% CI: 12.32–12.68). Incidence rates were highest in participants with loose teeth who were both hypertensive (IR: 15.90, 95% CI: 14.69–17.19) and non‐hypertensive (IR: 9.22, 95% CI: 8.12–10.43) (Table 2). The risk of CVD was increased more in hypertensive participants with PD compared to those without after adjustments (without PD—HR: 1.28, 95% CI: 1.25–1.32; PD – HR: 1.36, 95% CI: 1.21–1.53) (Figure 3).

### 3.4 Risk of depression

The rate of depression was similar in both non‐hypertensive and hypertensive healthy controls (IR: 2.18, 95% CI: 2.10–2.26; IR: 2.24, 95% CI: 2.21–2.32) (Table 2). Following adjustments, the risk of depression due to hypertension was not increased in healthy controls (HR: 0.96, 95% CI: 0.91–1.02) or participants with PD (HR: 0.95, 95% CI: 0.76–1.20) (Figure 3).

### 3.5 Risk of diabetes

The rate of diabetes in healthy controls was higher in participants who were hypertensive (IR: 8.42, 95% CI: 8.17–9.06) compared to non‐hypertensives (IR: 5.85, 95% CI: 5.71–5.98) (Table 2). Following adjustments, the risk of diabetes was higher in hypertensive healthy controls (HR: 1.19, 95% CI: 1.15–1.23) and marginally higher hypertensive participants with PD (HR: 1.11, 95% CI: 0.98–1.26) than in respective non‐hypertensives (Figure 3).

### 3.6 Risk of inflammatory disease

The rate of inflammatory disease in healthy controls was higher in participants who were hypertensive (IR: 14.64, 95% CI: 14.45–14.84) compared to non‐hypertensives (IR: 10.37, 95% CI: 10.19–10.55) (Table 2). After adjustments, hypertension did not increase the risk of inflammatory disease in healthy controls (HR: 0.99, 95% CI: 0.97–1.01) or in participants with PD (HR: 1.01, 95% CI: 0.91–1.13) (Figure 3).

### 3.7 Risk of renal disease

The rate of renal disease was lowest in non‐hypertensive healthy controls (IR: 2.05, 95% CI: 1.98–2.13), and non‐hypertensives with bleeding gums (IR: 1.82, 95% CI: 1.62–2.04) (Table 2). After adjustments, the risk of renal disease was increased in hypertensives with PD (HR: 1.35, 95% CI: 1.29–1.42) and without PD (HR: 1.24, 95% CI: 1.02–1.50) compared to non‐hypertensive participants (Figure 3).

### 3.8 Risk of respiratory disease

The overall rate of respiratory disease was increased in both hypertensive participants and those with PD (Figure 2). The risk of respiratory disease was not increased in hypertensive participants without PD (HR: 1.02, 95% CI: 0.98–1.06) compared, although there was a marginal increase in those with PD (HR: 1.11, 95% CI: 0.95–1.30) (Figure 3).

### 3.9 All‐cause mortality

The incidence of all‐cause mortality was highest in participants with loose teeth in both hypertension categories (non‐hypertensive IR: 4.85, 95% CI: 4.06–5.75; hypertensive IR: 7.50, 95% CI: 6.67–8.40) (Table 2). This risk was not increased in participants with PD when stratified by PD status (Figure 3).

Use of complete cases in the sensitivity analysis exhibited similar trends to those in the main analysis (see Supplementary Table S3 in online *Journal of Periodontology*). Removal of hypertensive participants with an antihypertensive prescription at baseline (*n* = 15,831) from the analysis also had similar effect to risk estimates (see Supplementary Table S4 in online *Journal of Periodontology*).

## 4 DISCUSSION

Our findings demonstrate that both PD and hypertension are associated with an increased risk of several subsequent systemic diseases. Overall, we found that hypertension as well as loose teeth and painful gums independently increase risk of several subsequent systemic diseases. In particular, hypertension had an additive effect with PD on risks of CVD and respiratory disease.

The findings show that participants with loose teeth (clinically indicative of severe PD) had the highest incidence of subsequent systemic diseases compared to the other oral health indicators. This reflects the growing evidence from studies that have shown an association of PD with development of a variety of chronic conditions including CVD, diabetes, and depression.^7^, ^22^, ^23^ The risk of all‐cause mortality was also increased in participants with loose teeth in the present study. Other findings using data from the UK Biobank cohort have shown that severe PD (specifically loose teeth) is associated with an increased risk of COVID‐19 mortality, as well as all‐cause mortality in people who are otherwise systemically healthy.^8^, ^10^

Our study is the first of its kind to explore the effect of both PD and hypertension on multiple subsequent systemic diseases. We previously identified the most frequent disease trajectories of PD in UK Biobank participants. Hypertension was central to several of the multimorbidity trajectories including other conditions such as CVD, diabetes, and respiratory disease.^10^ The present study found an increased risk of these conditions in participants with PD, with the highest rates observed in those with hypertension and loose teeth (severe PD) at baseline. We also found higher risks of diabetes, renal disease, respiratory disease, and all‐cause mortality in hypertensive participants with PD. This suggests that PD and hypertension have an additive effect on an individual's risk of developing subsequent systemic conditions.

Our findings indicate that the risk of further systemic disease development in hypertensives may be impacted by the severity of their PD condition. Higher risks of systemic diseases were observed in those that self‐reported loose teeth (clinically indicative of severe PD) than painful gums (moderate–severe PD). This finding is reflected in previous work showing augmented risks of adverse outcomes in loose teeth compared to the other PD indicators.^8^, ^10^, ^16^ PD can manifest across a spectrum of signs and symptoms; as such, the oral health indicators in the present study were selected to represent different severities of the disease: from painful gums (moderate PD) to loose teeth (severe PD). The intensified risks of systemic disease observed in hypertensive participants with painful gums and loose teeth could be reflective of the transference of inflammation: from localized inflammation in mild‐to‐moderate PD to generalized disease leading to attachment loss and tooth mobility in more severe cases.^5^ The systemic increase of circulating inflammatory cytokines results in global impaired endothelial cell function, thereby increasing arterial pressure.^24^ Periodontal inflammation has been linked to high blood pressure.^25^ One Mendelian randomization study has recently confirmed the causal association between PD and subsequent hypertension.^13^ The latter authors suggest that PD treatment may reduce inflammation in hypertensive participants,^13^ although the research regarding CRP as an inflammatory mediator between PD and hypertension is controversial.^12^, ^26^ A recent study also found that systemic inflammation only accounts for 10% of the mediating pathway effect that links PD with an increased risk of all‐cause mortality.^27^ Other findings suggest that PD treatment may also improve the efficacy of antihypertensive medication; although this study was limited to cross‐sectional data, and the impact to subsequent systemic disease outcomes was not explored.^28^ More robust research is needed to improve understanding of the biological mechanism that underpins the causal association between PD, hypertension, and systemic disease.

Our results also suggest that PD may independently increase the risk of CVD. People with PD have an increased risk of hypertension itself, suggesting that hypertension may precede further systemic disease diagnoses in this population.^10^, ^29^ One explanation is that the participants may have developed hypertension prior to a systemic disease diagnosis, and this diagnosis was not captured by the health outcomes data. Furthermore, we also used blood pressure measurements to determine hypertensive status; an arbitrary cutoff cannot account for participants that may have been borderline hypertensive at baseline and developed hypertension soon after. Complementary research is required to explore the possible mediator effect of raised blood pressure on the risk of subsequent systemic disease outcomes.

This novel study explored the impact of both hypertension and PD on subsequent systemic disease outcomes and as such has several strengths to note. Much of the previous research demonstrating independent associations of PD with hypertension and systemic diseases is based on cross‐sectional data or studies of smaller samples.^25^, ^26^, ^30^ The present study used “Big Data” from the UK Biobank longitudinal cohort and linked health outcomes.^14^ This allows for multiple outcomes of interest to be explored in a large cohort, as well as inferences on directionality of the observed associations to be suggested. The UK Biobank core dataset also enabled us to account for a number of risk factors that are independently associated with the conditions such as sex, BMI, and smoking status.^3^, ^4^ Another key strength to our study was the use of systemically healthy (with the exception of hypertension) population at baseline. This reduced possible confounding effects of other conditions and improved the reliability of risk estimates.

A limitation to our study is that the findings may not be generalizable to the whole population. The UK Biobank cohort consists of older adults, a major portion of which are of white ethnicity. While we are not able to apply our findings to younger people with PD, age is a known risk factor for PD and systemic diseases and has also been shown to be a mediating factor for severe PD and hypertension.^31^ Additionally, previous research has shown that PD is also more prevalent in ethnic minorities, and our results may therefore be an underestimate of the true associations in these at‐risk populations.^32^ Further research is required in this area to identify populations with PD who are most at risk for adverse systemic outcomes.

It should also be noted that the present study was limited by the use of self‐reported oral health indicators as surrogates for PD. There have been suggestions that self‐reported responses of loose teeth may also be associated with trauma or ageing, while mild cases of PD in smokers can also be difficult to identify.^15^ Of note, one systematic review concluded that self‐reported oral health indicators can be used as accurate case definitions of PD.^33^ The findings complement previous research of the systemic associations in people with PD, which could also suggest loose teeth as a viable case definition for severe cases.^7^, ^25^ Nonetheless, a clinical case definition of PD would eliminate the potential biases of self‐reported responses and provide more robust conclusions on the associations of PD severity. Furthermore, linked dental and health records would account for the effect of PD severity and subsequent PD treatment to an individual's risk of systemic disease.

## 5 CONCLUSION

Our study demonstrates the combined and individual effects of PD and hypertension on the risk of subsequent systemic disease. Overall, the effect of hypertension category was more impactful on estimates than PD status. The risks of CVD and respiratory disease were exacerbated in hypertensive participants with loose teeth (indicative of more severe PD). Shared inflammatory pathways and endothelial dysfunction are the probable cause in this association; however, the understanding of the biological mechanism that underpins the true causal associations between PD, hypertension, and subsequent systemic disease remains controversial and requires further research.

## FUNDING

Harriet Larvin was supported by the Hopper Scholarship at the University of Leeds. The research is supported by the National Institute for Health and Care Research (NIHR) infrastructure at Leeds. The views expressed are those of the author(s) and not necessarily those of the NHS, the NIHR, or the Department of Health and Social Care.

## CONFLICT OF INTEREST

The authors report no conflicts of interest related to this study.

## AUTHOR CONTRIBUTIONS

All authors have made substantial contributions to conception and design of the study. Harriet Larvin contributed to conception, data analysis, and interpretation, and drafted the manuscript. Jing Kang, Vishal R. Aggarwal, and Sue Pavitt contributed to interpretation and critically revised the manuscript. Jianhua Wu contributed to conception, study design, data acquisition, and interpretation, and critically revised the manuscript. All authors were involved in writing the paper and had final approval of the submitted and published versions.

## Supporting information


**Supplemental Table 1**. ICD‐10 and Read (v2) codes used for identifying systemic diseases.

**Supplemental Table 2**. Antihypertensive medications.

**Supplemental Table 3**. Association between oral health indicators and risk of subsequent systemic disease compared to healthy controls, stratified by hypertension category and using complete cases only.

**Supplemental Table 4**. Association between oral health indicators and risk of subsequent systemic disease compared to healthy controls and excluding those on antihypertensive medication, stratified by hypertension category.
