# Supplementary material for: The additive effect of periodontitis with hypertension on risk of systemic disease and mortality
Source: J Periodontol. 2022 May 27;93(7):1024–35. doi: 10.1002/JPER.21-0621 (PMC9544472; doi:10.1002/JPER.21-0621)
Supplement: Supplementary file 1 — Supplemental Table 1. ICD‐10 and Read (v2) codes used for identifying systemic diseases. [file JPER-93-1024-s002.docx]

**Supplemental Table 1** ICD-10 and Read (v2) codes used for identifying systemic diseases.

| **Systemic Disease** | **ICD-10 Codes** | **Read (v2) Codes** |
| --- | --- | --- |
| Cancers (all) | C00,C01,C02,C03,C04,C05,C06,C07,C08,C09,C10,C11,C12,C13,C14,C15,C16,C17,C18,C19,C20,C21,C22,C23,C24,C25,C26,C30,C31,C32,C33,C34,C37,C38,C39,C40,C41,C43,C45,C46,C47,C48,C49,C50,C51,C52,C53,C54,C55,C56,C57,C58,C60,C61,C62,C63,C64,C65,C66,C67,C68,C69,C70,C71,C72,C73,C74,C75,C76,C80,C97,C900,C901,C910,C911,C913,C916,C917,C918,C919,C92,C93,C940,C942,C943,C947,C95,C81,C82,C83,C84,C85,C883,C887,C889,C914,C915,C96,C77,C78,C79,C86,C880,C882,C884,C902,C903,C944,C946,C44 | 142..00,142..11,142..12,142..13,142..15,1421,1422,1423,1423.11,1423.12,1424,1425,1425000,1426,1427,1428,1428.11,1428.12,1429,142A.00,142B.00,142Z.00,ZV10.00,ZV10000,ZV10011,ZV10012,ZV10014,ZV10015,ZV10016,ZV10017,ZV10018,ZV10100,ZV10111,ZV10112,ZV10200,ZV10211,ZV10212,ZV10214,ZV10300,ZV10400,ZV10411,ZV10414,ZV10415,ZV10416,ZV10417,ZV10500,ZV10511,ZV10512,ZV10513,ZV10600,ZV10611,ZV10711,ZV10y00,ZV10y11,ZV10y12,ZV10y14,ZV10y15,ZV10y16,ZV10z00,ZV67600,ZV67700,ZV67800,ZV67811,ZV67900,ZV67A00,ZV67B00,1J0..00,1J00.00,1J01.00,1J02.00,1J03.00,1J04.00,1J06.00,1J07.00,1J08.00,1J09.00,1J0A.00,1J0B.00,1J0C.00,1J0D.00,1J0E.00,1J0F.00,1J0G.00,1J0H.00,1J0I.00,1J0J.00,1J0K.00,8HH8.00,8HHt.00,8Hn..00,8Hn0.00,8Hn1.00,8Hn2.00,8Hn3.00,8Hn4.00,8Hn5.00,8Hn6.00,8Hn7.00,8Hn8.00,8Hn9.00,8HnA.00,8HnB.00,ZV71100,1O0..00,4M00.00,4M01.00,4M02.00,4M72.00,7G03J00,7G03K00,8B3p.00,8BAD000,8BC6.00,8BCF.00,A788W00,A789500,B....11,B0...00,B0...11,B00..00,B00..11,B000.00,B000000,B000100,B000z00,B001.00,B001000,B001100,B002200,B002300,B003.00,B003000,B003100,B003200,B003300,B003z00,B004.00,B004000,B004200,B005.00,B006.00,B007.00,B00z100,B00zz00,B01..00,B010.00,B010.11,B010000,B010z00,B011.00,B011z00,B012.00,B013.00,B013100,B013z00,B014.00,B015.00,B016.00,B017.00,B01y.00,B01z.00,B02..00,B020.00,B021.00,B022.00,B02y.00,B02z.00,B03..00,B030.00,B031.00,B03z.00,B04..00,B040.00,B041.00,B042.00,B04y.00,B04z.00,B05..00,B050.00,B050.11,B051000,B051100,B052.00,B053.00,B054.00,B055.00,B055000,B055100,B055z00,B056.00,B057.00,B05y.00,B05z.00,B05z000,B06..00,B060.00,B060000,B060z00,B061.00,B062.00,B062000,B062200,B062300,B063.00,B064.00,B064000,B064100,B064z00,B065.00,B066.00,B067.00,B06y.00,B06yz00,B06z.00,B07..00,B070.00,B071.00,B071000,B071100,B071z00,B072.00,B072000,B073.00,B074.00,B07y.00,B07z.00,B08..00,B080.00,B081.00,B082.00,B083.00,B08y.00,B08z.00,B0z..00,B0z0.00,B0z1.00,B0z2.00,B0zy.00,B0zz.00,B1...00,B1...11,B10..00,B100.00,B101.00,B102.00,B103.00,B104.00,B105.00,B106.00,B107.00,B10y.00,B10z.00,B10z.11,B11..00,B11..11,B110.00,B110100,B110111,B110z00,B111.00,B111000,B111100,B111z00,B112.00,B113.00,B114.00,B115.00,B116.00,B117.00,B118.00,B119.00,B11y.00,B11y000,B11y100,B11yz00,B11z.00,B12..00,B120.00,B121.00,B122.00,B123.00,B124.00,B12z.00,B13..00,B130.00,B131.00,B132.00,B133.00,B134.00,B134.11,B135.00,B136.00,B137.00,B138.00,B13y.00,B13z.00,B13z.11,B14..00,B140.00,B141.00,B141.11,B141.12,B142.00,B142.11,B142000,B143.00,B14y.00,B14z.00,B15..00,B150.00,B150000,B150100,B150200,B150300,B150z00,B151.00,B151000,B151200,B151400,B151z00,B152.00,B15z.00,B16..00,B160.00,B160.11,B161.00,B161000,B161100,B161200,B161211,B161z00,B162.00,B163.00,B16y.00,B16z.00,B17..00,B170.00,B171.00,B172.00,B173.00,B174.00,B175.00,B17y.00,B17y000,B17yz00,B17z.00,B18..00,B180.00,B180100,B180200,B180z00,B181.00,B18y.00,B18y100,B18y200,B18y300,B18y400,B18y500,B18y600,B18y700,B18yz00,B18z.00,B1z..00,B1z0.00,B1z0.11,B1z1.00,B1z1100,B1z1z00,B1z2.00,B1zy.00,B1zz.00,B2...00,B2...11,B20..00,B200.00,B200000,B200200,B200300,B200z00,B201.00,B201100,B201200,B201300,B201z00,B202.00,B203.00,B204.00,B205.00,B206.00,B20y.00,B20z.00,B21..00,B210.00,B211.00,B212.00,B213.00,B213000,B213100,B213300,B213z00,B214.00,B215.00,B21y.00,B21z.00,B22..00,B220.00,B220z00,B221.00,B221000,B221100,B221z00,B222.00,B222.11,B222000,B222100,B222z00,B223.00,B223000,B223100,B223z00,B224.00,B224000,B224100,B224z00,B225.00,B226.00,B22y.00,B22z.00,B22z.11,B23..00,B230.00,B232.00,B23y.00,B23z.00,B24..00,B240.00,B241.00,B241000,B241200,B241300,B241z00,B242.00,B243.00,B24X.00,B24z.00,B25..00,B26..00,B2z..00,B2z0.00,B2zy.00,B2zz.00,B3...00,B3...11,B3...12,B30..00,B300.00,B300000,B300100,B300200,B300300,B300400,B300500,B300600,B300700,B300800,B300900,B300A00,B300B00,B300C00,B300z00,B301.00,B302.00,B302000,B302100,B302200,B302z00,B303.00,B303000,B303100,B303200,B303300,B303400,B303500,B303z00,B304.00,B304000,B304200,B304300,B304400,B304z00,B305.00,B305.12,B305000,B305100,B305C00,B305D00,B305z00,B306.00,B306000,B306100,B306200,B306300,B306400,B306500,B306z00,B307.00,B307000,B307100,B307200,B307z00,B308100,B308200,B308300,B308800,B308B00,B308D00,B30W.00,B30X.00,B30z.00,B30z000,B31..00,B310.00,B310000,B310100,B310200,B310300,B310400,B310z00,B311.00,B311000,B311100,B311200,B311300,B311400,B311500,B312.00,B312100,B312200,B312300,B312400,B312z00,B313.00,B313000,B313100,B313200,B313z00,B314.00,B314000,B314100,B314z00,B315.00,B315000,B315100,B315200,B315z00,B316.00,B31y.00,B31z.00,B32..00,B320.00,B321.00,B322.00,B322000,B322z00,B323.00,B323000,B323100,B323200,B323300,B323400,B323500,B323z00,B324.00,B324000,B324100,B325.00,B325000,B325100,B325200,B325300,B325500,B325600,B325700,B325800,B325z00,B326.00,B326000,B326100,B326200,B326300,B326400,B326500,B326z00,B327.00,B327000,B327100,B327200,B327300,B327400,B327500,B327600,B327700,B327800,B327900,B327z00,B32y.00,B32y000,B32z.00,B331000,B331100,B331200,B332200,B339.00,B33z000,B34..00,B34..11,B340.00,B340000,B340100,B340z00,B341.00,B342.00,B343.00,B344.00,B345.00,B346.00,B347.00,B34y.00,B34y000,B34yz00,B34z.00,B35..00,B350.00,B350000,B350100,B35z.00,B35z000,B35zz00,B3y..00,B3z..00,B4...00,B4...11,B40..00,B41..00,B41..11,B410.00,B410000,B410100,B410z00,B411.00,B412.00,B41y.00,B41y000,B41y100,B41yz00,B41z.00,B42..00,B420.00,B43..00,B430.00,B430000,B430100,B430200,B430211,B430300,B430z00,B431.00,B431000,B431z00,B432.00,B43y.00,B43z.00,B44..00,B440.00,B440.11,B441.00,B443.00,B44y.00,B44z.00,B45..00,B450.00,B450100,B450z00,B451.00,B451000,B451z00,B452.00,B453.00,B454.00,B454.11,B45X.00,B45y.00,B45y000,B45z.00,B46..00,B47..00,B470.00,B470200,B470300,B470z00,B471.00,B471000,B471100,B471z00,B47z.00,B47z.11,B47z.12,B48..00,B480.00,B481.00,B482.00,B483.00,B484.00,B485.00,B486.00,B487.00,B48y.00,B48y000,B48y100,B48y200,B48yz00,B48z.00,B49..00,B490.00,B491.00,B492.00,B493.00,B494.00,B495.00,B496.00,B497.00,B49y.00,B49y000,B49z.00,B4A..00,B4A..11,B4A0.00,B4A0000,B4A1.00,B4A1000,B4A1z00,B4A2.00,B4A3.00,B4A4.00,B4Ay.00,B4Ay000,B4Az.00,B4y..00,B4z..00,B5...00,B5...11,B50..00,B500.00,B500000,B500100,B500z00,B501.00,B501000,B501z00,B502.00,B503.00,B504.00,B505.00,B506.00,B507.00,B507100,B508.00,B50y.00,B50z.00,B51..00,B51..11,B510.00,B510000,B510100,B510400,B510500,B510z00,B511.00,B512.00,B512000,B512z00,B513.00,B514.00,B515.00,B515000,B516.00,B517.00,B517000,B517100,B517200,B517300,B517z00,B51y.00,B51y000,B51y200,B51z.00,B52..00,B520000,B520100,B520200,B521.00,B521z00,B522.00,B523.00,B523z00,B524.00,B524000,B524100,B524200,B524300,B524400,B524500,B524600,B525.00,B52W.00,B52X.00,B52y.00,B52z.00,B53..00,B54..00,B540.00,B540.11,B540000,B540100,B540z00,B541.00,B542.00,B542000,B542100,B542z00,B543.00,B544.00,B545.00,B545000,B545100,B545200,B54X.00,B54y.00,B54z.00,B55..00,B550.00,B550000,B550100,B550200,B550300,B550400,B550500,B550z00,B551.00,B551000,B551100,B551200,B551z00,B552.00,B553.00,B553000,B553100,B553z00,B554.00,B555.00,B55y.00,B55y000,B55y100,B55y200,B55yz00,B55z.00,B57..12,B576200,B58..11,B59..00,B591.00,B592.00,B592X00,B593.00,B59z.00,B59zX00,B5y..00,B5z..00,B6z0.00,B7F1000,B7F2000,B7F4000,B830.00,B911013,B933.11,BB02.00,BB07.00,BB08.00,BB09.00,BB0A.00,BB11.11,BB12.00,BB13.11,BB17.00,BB18.00,BB19.00,BB1A.00,BB1B.00,BB1C.00,BB1D.00,BB1E.00,BB1F.00,BB1G.00,BB1J.00,BB1J.12,BB1K.00,BB1L.00,BB1M.00,BB1N.00,BB22.00,BB24.00,BB24.11,BB26.11,BB29.12,BB2A.11,BB2A.12,BB2C.11,BB2M.00,BB35.00,BB36.00,BB4..00,BB43.00,BB43.11,BB47.00,BB48.00,BB49.00,BB4A.00,BB4z.00,BB5..00,BB5..11,BB52.00,BB52000,BB54.00,BB55.00,BB56.00,BB57.00,BB58.00,BB5B.00,BB5B100,BB5B600,BB5Bz00,BB5C.00,BB5Cz00,BB5D.00,BB5D.11,BB5D100,BB5D111,BB5D300,BB5D500,BB5D513,BB5D800,BB5Dz00,BB5F.00,BB5J.00,BB5J.11,BB5K.00,BB5L.00,BB5L100,BB5L300,BB5Lz00,BB5M.00,BB5M100,BB5Mz00,BB5N.00,BB5N.11,BB5N100,BB5Nz00,BB5P.00,BB5R100,BB5R600,BB5R900,BB5RA00,BB5S.00,BB5S200,BB5S211,BB5S212,BB5S400,BB5Sz00,BB5T.00,BB5T100,BB5Tz00,BB5U.00,BB5U100,BB5U200,BB5Uz00,BB5V.00,BB5V100,BB5V311,BB5V700,BB5V711,BB5Vz00,BB5W.00,BB5W100,BB5W111,BB5W112,BB5Wz00,BB5X.00,BB5X100,BB5Xz00,BB5a.00,BB5a000,BB5a012,BB5az00,BB5b.00,BB5c.00,BB5cz00,BB5f.00,BB5f100,BB5f111,BB5f200,BB5f300,BB5f600,BB5f700,BB5fz00,BB5h.00,BB5h100,BB5hz00,BB5j.00,BB5j200,BB5jz00,BB5y000,BB5z.00,BB60.00,BB60100,BB61.00,BB61200,BB62.00,BB62100,BB69.00,BB69100,BB69z00,BB6A.00,BB71.00,BB80.00,BB80100,BB80z00,BB81.11,BB81200,BB81500,BB81800,BB81B00,BB81E00,BB81E11,BB81H00,BB82.00,BB82100,BB82111,BB82112,BB82114,BB82z00,BB84.00,BB85.00,BB85000,BB85z00,BB90.00,BB91.00,BB91.11,BB91000,BB91100,BB92.00,BB93.00,BB94.00,BB94.11,BB9B.00,BB9B.11,BB9C.00,BB9F.00,BB9G.00,BB9H.00,BB9K.00,BB9K000,BB9M.00,BBA2.00,BBB0.00,BBB2.00,BBB3.00,BBB4.00,BBB5.00,BBB7.00,BBC4.00,BBCA.00,BBCC100,BBD1.00,BBDB.00,BBE..00,BBE1.00,BBE1.11,BBE1.12,BBE1.13,BBE1.14,BBE1000,BBE1100,BBE2.00,BBE4.00,BBEA.00,BBEC.00,BBED.00,BBEG.00,BBEG.11,BBEG000,BBEH.00,BBEM.00,BBEN.11,BBEP.00,BBEQ.00,BBES.00,BBET.00,BBEz.00,BBF..00,BBF1.00,BBF2.00,BBF3.00,BBF4.00,BBF4.11,BBF5.00,BBF5.11,BBF6.00,BBFz.00,BBG1.00,BBG3.00,BBG8.00,BBG8.11,BBGJ.11,BBGM.00,BBGP.00,BBH1.00,BBJ1.00,BBJ3.00,BBJ5.00,BBJ5.12,BBJ7.00,BBJ8.00,BBJH.00,BBK0200,BBK0400,BBK0700,BBK1100,BBK2.00,BBK2100,BBK2z00,BBK3100,BBK3200,BBK3600,BBK3611,BBK3700,BBL..00,BBL0.00,BBL4.00,BBL7.00,BBL7111,BBL8.00,BBL9.00,BBLA.00,BBLD.00,BBLE.00,BBLG.00,BBLH.00,BBLJ.00,BBLz.00,BBM0100,BBM8.00,BBM9.00,BBN1.00,BBN4.00,BBN5.00,BBP1.00,BBP5.00,BBP7.00,BBP9.00,BBPX.00,BBQ1.00,BBQ1000,BBQ1100,BBQ1z00,BBQ3.00,BBQ7.00,BBQ7011,BBQ7012,BBQ7100,BBQ7200,BBQ7211,BBQ7212,BBQ7300,BBQ7400,BBQ7500,BBQ7z00,BBR2.00,BBR3.00,BBR4.00,BBT1.00,BBT1.11,BBTA.00,BBU1.00,BBV..00,BBV..12,BBV1.00,BBV1.11,BBV1.12,BBV1.13,BBV2.00,BBV3.00,BBV4.00,BBV5.00,BBV9.00,BBVA.00,BBVz.00,BBW4.00,BBW4.11,BBW6.00,BBW9.00,BBX1.00,BBX1.11,BBY0.00,BBY0.11,BBZ2.00,BBZ2.11,BBZC.00,BBZN.00,BBZN.11,BBb0.00,BBb0.11,BBb0.12,BBb5.00,BBbW.00,BBbz.00,BBcC.00,BBcC.11,BBd2.11,BBd2.12,BBe2.00,BBe9.00,BBf..00,BBf2.00,BBh0.11,Byu0.00,Byu1.00,Byu1100,Byu1200,Byu1300,Byu2.00,Byu2000,Byu2100,Byu2400,Byu2500,Byu3.00,Byu3100,Byu3200,Byu3300,Byu4.00,Byu4000,Byu4100,Byu5.00,Byu5000,Byu5011,Byu5100,Byu5300,Byu5700,Byu5800,Byu5900,Byu5B00,Byu6.00,Byu7.00,Byu7000,Byu7100,Byu7300,Byu8.00,Byu8000,Byu8200,Byu9.00,Byu9000,ByuA.00,ByuA000,ByuA100,ByuA200,ByuA300,ByuB.00,ByuB100,ByuC.00,ByuC000,ByuC100,ByuC800,ByuE.00,ByuE000,D212000,B630.00,B630.11,B630.12,B630000,B630100,B630200,B630300,B631.00,B64..00,B64..11,B640.00,B641.00,B641.11,B642.00,B64y.00,B64y100,B64yz00,B64z.00,B65..00,B650.00,B651.00,B651.11,B651z00,B652.00,B653.00,B653000,B653100,B65y100,B65yz00,B65z.00,B66..00,B66..12,B660.00,B661.00,B66z.00,B67..00,B670.00,B670.11,B672.00,B672.11,B673.00,B674.00,B675.00,B67y.00,B67z.00,B68..00,B680.00,B681.00,B682.00,B68y.00,B68z.00,B69..00,B690.00,B691.00,B6y0.00,B6y0.11,B6y1.00,B936.11,B936.12,BBn..00,BBn0.00,BBn0.11,BBn0.12,BBn0.13,BBn0.14,BBn2.00,BBn2.12,BBnz.00,BBr..00,BBr0.00,BBr0000,BBr0100,BBr0111,BBr0112,BBr0113,BBr0200,BBr0300,BBr0400,BBr0z00,BBr2.00,BBr2000,BBr2011,BBr2100,BBr2300,BBr2500,BBr3.00,BBr4.00,BBr4000,BBr6.00,BBr6000,BBr6011,BBr6100,BBr6300,BBr6311,BBr6600,BBr6700,BBr6800,BBr6z00,BBr8.00,BBr8000,BBr9000,BBrA.00,BBrA100,BBrA111,BBrA300,BBrA311,BBrA312,BBrA500,BBrA700,BBrz.00,BBs..00,BBs2.00,BBsz.00,ByuD500,ByuD600,ByuD700,ByuD800,ByuD900,N330900,4M20.00,4M21.00,4M22.00,4M23.00,A789600,A789700,AyuC600,B6...00,B6...11,B60..00,B600.00,B600000,B600100,B600300,B600700,B601.00,B601000,B601100,B601200,B601300,B601z00,B602.00,B602100,B602300,B602500,B602z00,B61..00,B610.00,B610300,B611.00,B612.00,B612400,B613.00,B613000,B613100,B613200,B613300,B613500,B613600,B613z00,B614.00,B614000,B614100,B614200,B614300,B614400,B614800,B614z00,B615.00,B615000,B615100,B615200,B615z00,B616.00,B616000,B616400,B61z.00,B61z000,B61z100,B61z200,B61z400,B61z700,B61z800,B61zz00,B62..00,B620.00,B620000,B620100,B620300,B620500,B620800,B620z00,B621.00,B621000,B621300,B621400,B621500,B621800,B621z00,B622.00,B623.00,B623000,B623100,B623300,B623z00,B624.00,B624.11,B624.12,B624000,B624300,B624z00,B625.00,B625.11,B625800,B625z00,B626.00,B626800,B626z00,B627.00,B627000,B627100,B627200,B627300,B627500,B627600,B627700,B627800,B627900,B627B00,B627C00,B627C11,B627D00,B627W00,B627X00,B62x.00,B62x000,B62x100,B62x200,B62x400,B62x500,B62x600,B62xX00,B62y.00,B62y000,B62y100,B62y200,B62y300,B62y400,B62y500,B62y600,B62y700,B62y800,B62yz00,B62z.00,B62z100,B62z200,B62z500,B62zz00,B62zz11,B63..00,B63y.00,B63z.00,B64y200,B67y000,B6y..00,B6z..00,BBB1.00,BBg..00,BBg1.00,BBg1.11,BBg1000,BBg2.00,BBg2.11,BBg3.00,BBg4.00,BBg5.00,BBg7.00,BBg8.00,BBgA.00,BBgB.00,BBgC.00,BBgC.11,BBgC.12,BBgD.00,BBgE.00,BBgG.00,BBgG.11,BBgG.12,BBgH.00,BBgJ.00,BBgK.00,BBgL.00,BBgM.00,BBgN.00,BBgP.00,BBgQ.00,BBgR.00,BBgS.00,BBgT.00,BBgV.00,BBgz.00,BBh0.00,BBj..00,BBj0.00,BBj1.00,BBj1000,BBj1100,BBj2.00,BBj4.00,BBj6.00,BBj6000,BBj6100,BBj6200,BBj9.00,BBjz.00,BBk..00,BBk0.00,BBk0.12,BBk0.13,BBk7.00,BBkz.00,BBl..00,BBl0.00,BBl1.00,BBm1.00,BBm1.11,BBm3.12,BBm4.00,BBm5.00,BBm9.00,BBmD.00,BBmH.00,BBp1.00,BBr2600,BBr2700,BBrA400,BBv0.00,BBv2.00,ByuD.00,ByuD000,ByuD100,ByuD200,ByuD300,ByuDB00,ByuDC00,ByuDE00,ByuDF00,ByuDF11,C37y000,C37y100,C37y500,C37y600,C37yB00,1D18.00,5136,B153.00,B56..00,B56..11,B560.00,B560000,B560100,B560200,B560300,B560400,B560500,B560600,B560700,B560800,B560900,B560z00,B561.00,B561000,B561200,B561300,B561400,B561500,B561600,B561700,B561800,B561900,B561z00,B562.00,B562000,B562100,B562200,B562300,B562400,B562z00,B563.00,B563000,B563100,B563200,B563300,B563z00,B564.00,B564000,B564100,B564z00,B565.00,B565000,B565300,B565z00,B56y.00,B56z.00,B57..00,B57..11,B570.00,B571.00,B572.00,B573.00,B574.00,B574000,B574z00,B575.00,B575000,B575100,B575z00,B576.00,B576000,B576100,B576z00,B577.00,B577.11,B57y.00,B57z.00,B58..00,B580.00,B581.00,B581000,B581100,B581200,B581z00,B582.00,B582000,B582200,B582300,B582400,B582500,B582600,B582z00,B583.00,B583000,B583100,B583200,B583z00,B584.00,B585.00,B585000,B586.00,B587.00,B58y.00,B58y000,B58y100,B58y200,B58y211,B58y300,B58y400,B58y411,B58y500,B58y600,B58y700,B58y900,B58yz00,B58z.00,B590.00,B590.11,B594.00,BB03.00,BB03.11,BB04.00,BB13.00,BB14.00,BB2B.00,BB53.00,BB85100,ByuC200,ByuC300,ByuC400,ByuC500,ByuC600,ByuC700,BBmK.00,C330000,C333.00,C333000,C333z00,D41y100,7G05D00,B33..00,B33..11,B33..14,B33..15,B330.00,B331.00,B332.00,B332000,B332100,B332z00,B333.00,B333000,B333100,B333200,B333300,B333400,B333500,B333z00,B334.00,B334000,B334100,B334z00,B335.00,B335000,B335100,B335200,B335300,B335400,B335500,B335600,B335700,B335800,B335900,B335A00,B335z00,B336.00,B336000,B336100,B336200,B336300,B336400,B336500,B336z00,B337.00,B337000,B337100,B337200,B337300,B337400,B337500,B337700,B337800,B337900,B337z00,B338.00,B33X.00,B33y.00,B33z.00,B33z.11,B33z100,BB24.12,BB26.00,BB29.13,BB2A.00,BB2A.13,BB2C.00,BB2D.00,BB2E.00,BB2F.00,BB2G.00,BB2J.00,BB31.00,BB32.00,BB33.00,BB34.00,Byu4200,Byu4300,Byu5A00 |
| Hypertension | I10,I11,I12,I13,I15 | 14A2.00,2126100,212K.00,9OI9.00,1JD..00,246M.00,662..12,6629,662H.00,662P.00,8CR4.00,9N03.00,9N1y200,9N4L.00,9OI..00,9OI..11,9OI1.00,9OI2.00,9OI3.00,9OI4.00,9OI5.00,9OI6.00,9OI7.00,9OI8.00,9OIA.00,9OIA.11,9OIZ.00,9h3..00,9h31.00,9h32.00,6624,6627,6628,662F.00,662G.00,662O.00,662b.00,662c.00,662d.00,662r.00,7Q01.00,8B26.00,8BL0.00,8I3N.00,F404200,F421300,G2...00,G2...11,G20..00,G200.00,G201.00,G202.00,G203.00,G20z.00,G20z.11,G21..00,G210.00,G210000,G210100,G211.00,G211000,G211100,G21z.00,G21z000,G21z011,G21z100,G21zz00,G22..00,G220.00,G221.00,G222.00,G22z.00,G22z.11,G23..00,G230.00,G231.00,G232.00,G233.00,G234.00,G23z.00,G2y..00,G2z..00,G672.00,G672.11,Gyu2.00,L122.00,L122000,L122100,L122300,L122z00,L127.00,L127z00,L128.00,L128000,L128200,TJC7.00,TJC7z00,U60C500,U60C511,U60C51A,6146200,G24..00,G240.00,G240000,G240z00,G241.00,G241000,G241z00,G244.00,G24z.00,G24z000,G24z100,G24zz00,Gyu2100 |
| Cardiovascular diseases including:  atrial fibrillation, myocardial infarction, peripheral vascular disease, coronary heart disease, hypertension or angina, stroke or transient ischemic attack. | I201,I208,I209,I200,I472,I490,I460,I469,I470,I252,I21,I22,I23,I241,G458,G459,I690,I61,I60,I620,I621,I629,I693,I63,I691,I692,I694,I698,G463,G464,G465,G466,G467,I70,I713,I714,I715,I716,I718,I719,I710,I711,I712,I72,I731,I738,I739,I743,I744,I745,I730, I110,I130,I132,I260,I50,I48 | 14AB.00,G65z000,G65z100,Fyu5500,G65..00,G65..12,G65y.00,G65z.00,G65zz00,182..00,1822,1823,1824,1826,1828,1829,182B000,182C.00,182Z.00,8HTG.00,8HTJ.00,9N0f.00,R065.00,R065000,R065011,R065100,R065200,R065600,R065700,R065800,R065900,R065C00,R065D00,R065z00,Ryu0400,182A.00,1825,1827,1827.11,182B.00,R065300,R065400,R065500,R065A00,R065B00,R065B14,G33z400,G311500,14AP.00,14AD.00,7937600,793F200,793F300,793F400,3282,G571.11,3283,7L1H.13,G574.00,G574000,G574011,G574z00,7937500,793F.00,793F000,793F100,793F500,793Fy00,793Fz00,2241,7932111,7L1H600,853..00,8531,8532,8532.11,853Z.00,G575.00,G575.11,G575.12,G575000,G575200,G575300,G575z00,SP11000,14A3.00,14A4.00,14AH.00,G310.00,G32..00,G32..11,G32..12,G33z500,G30..11,G30..14,G30..17,G30A.00,G30X000,G307100,323..00,3233,3234,3235,3236,323Z.00,889A.00,G30..00,G30..12,G30..13,G30..15,G30..16,G300.00,G301.00,G301000,G301100,G301z00,G302.00,G303.00,G304.00,G305.00,G306.00,G307.00,G307000,G308.00,G309.00,G30B.00,G30X.00,G30y.00,G30y000,G30y100,G30y200,G30yz00,G30z.00,G31y100,G38..00,G380.00,G381.00,G384.00,G38z.00,Gyu3400,G35..00,G350.00,G351.00,G353.00,G35X.00,G310.11,G36..00,G360.00,G361.00,G362.00,G363.00,G364.00,G365.00,G366.00,G501.00,662o.00,G681.00,G682.00,G61..00,G61..11,G61..12,G610.00,G611.00,G612.00,G613.00,G614.00,G616.00,G617.00,G618.00,G61X.00,G61X000,G61X100,G61z.00,Gyu6200,Gyu6F00,G601.00,G602.00,G60X.00,7017000,G621.00,G622.00,G623.00,S62..13,S622.00,S623.00,S628.00,S629.00,S629000,S629100,7032000,G620.00,S62..11,S624.00,S624.11,S625.00,S626.00,S62A.00,G62..00,G62z.00,A94y600,S62..00,S62..14,S62z.00,S63..00,S63z.00,14A7.00,14A7.11,14A7.12,14AK.00,662e.00,7P24200,8HHM.00,9Om..00,9Om0.00,9Om1.00,9Om2.00,9Om3.00,9Om4.00,G68X.00,ZV12511,ZV12512,13YA.00,38DM.11,662M.00,9N0p.00,9N4X.00,9h2..00,9h21.00,9h22.00,C315100,Fyu5700,G671000,ZLEP.00,Fyu5600,G64z100,G64z111,G66..00,G66..11,G66..12,G66..13,G663.00,G664.00,G665.00,G666.00,G667.00,G668.00,L440.11,L440.12,G683.00,G64..11,G64..13,G671.00,G6W..00,G6X..00,Gyu6300,Gyu6400,Gyu6500,Gyu6600,Gyu6G00,14AE.00,14NB.00,16I..00,38DJ.00,662U.00,9N4h.00,G5y2.00,G70..00,G70..11,G700.00,G701.00,G703.00,G70y.00,G70y000,G70z.00,P769000,G71..00,G713.00,G713.11,G713000,G714.00,G714.11,G714000,G715.00,G715000,G716.00,G716000,G718.00,G71z.00,G710.00,G673200,G711.00,G711.11,G712.00,G720.00,G720000,G720100,G720200,G720z00,G721.00,G722.00,G722000,G722100,G722200,G722z00,G723.00,G723000,G723100,G723200,G723300,G723400,G723500,G723z00,G72y.00,G72y000,G72y100,G72y200,G72y400,G72y600,G72yB00,Gyu7300,2G63.00,A3A0F00,C107.00,C107000,C107100,C107300,C107400,C107z00,C108G00,C109F00,C109F11,C109F12,C10EG00,C10FF00,G700.11,G702.00,G702z00,G73..00,G73..11,G73..12,G73..13,G731.00,G731000,G731z00,G732.00,G732000,G732100,G733.00,G73y.00,G73y000,G73y100,G73y200,G73y400,G73y500,G73y511,G73y600,G73y700,G73y800,G73yz00,G73z.00,G73z000,G73z011,G73zz00,G740.12,G742z00,Gyu7400,M271.12,M271000,M271300,M271400,R054200,R054300,R055000,R055011,G742400,G742500,G742600,G742700,G742900,G74y000,G74y100,G74y200,G74y300,G730.00,G730000,G730100,G730z00,14A6.00,14AM.00,1736,1J60.00,23E1.00,388D.00,662T.00,662f.00,662g.00,662h.00,662i.00,679X.00,8CL3.00,8HBE.00,8HHz.00,8Hg8.00,8Hk0.00,9N0k.00,9N2p.00,9N4s.00,9N4w.00,9N6T.00,9On..00,9On0.00,9On1.00,9On2.00,9On3.00,9On4.00,9Or..00,9Or1.00,9Or2.00,9Or3.00,9Or4.00,9Or5.00,9h1..00,9h11.00,9h12.00,9hH..00,9hH0.00,9hH1.00,G581.12,G58z.11,H54..00,H541.00,H541000,H541z00,H54z.00,H584.00,H584z00,ZRad.00,G580400,G210.00,G210000,G210100,G211100,G21z100,G230.00,G232.00,G234.00,G1yz100,1O1..00,662W.00,662p.00,8B29.00,8H2S.00,9Or0.00,G400.00,G41z.11,G554000,G554011,G58..00,G58..11,G580.00,G580.11,G580.12,G580.13,G580.14,G580000,G580100,G580200,G580300,G581.00,G581.11,G581.13,G581000,G582.00,G58z.00,G58z.12,G5yy900,G5yyA00,R2y1000,Q48y100,14AN.00,14AR.00,212R.00,662S.00,6A9..00,9Os..00,9Os0.00,9Os1.00,9Os2.00,9Os3.00,9Os4.00,9hF..00,9hF1.00,G573200,G573400,G573500,3272,G573000,G573300,G573.00,G573z00,3273,G573100 |
| Respiratory diseases including:  chronic obstructive pulmonary disorder, asthma, bronchitis, bronchieactasis | J00,J01,J02,J03,J04,J05,J06,J07,J08,J09,J10,J11,J12,J13,J14,J15,J16,J17,J18,J19 | 14B3.11,14OJ.00,1J71.00,66Yg.00,679V.00,8CE6.00,H060000,H060200,H060v00,H06z200,H30..12,H300.00,H301.00,H302.00,H30z.00,H310.00,H310000,H310z00,H311100,H311z00,H312.00,H312000,H312011,H313.00,H31y.00,H31y100,H31yz00,H32y000,H32y100,H32y111,H32y200,H3y..00,H581.00,H582.00,66YB.00,66YD.00,66YL.00,66YM.00,66YS.00,66YT.00,9Oi..00,9Oi0.00,9Oi1.00,9Oi2.00,H3...00,H3...11,H31..00,H312100,H312z00,H31z.00,H32..00,H320.00,H320000,H320100,H320200,H320z00,H321.00,H322.00,H32y.00,H32yz00,H32z.00,H36..00,H37..00,H38..00,H39..00,H3y..11,H3z..00,H3z..11,Hyu3000,Hyu3100,66Yf.00,H06..00,H060.00,H060.11,H060300,H060400,H060500,H060600,H060700,H060800,H060900,H060A00,H060B00,H060C00,H060D00,H060E00,H060F00,H060w00,H060x00,H060z00,H06z.00,H06z000,H06z011,H20..11,H21..11,H22..11,H23..11,H24..11,H25..11,H26..11,H270.11,H30..00,H30..11,H311.00,H311000,Hyu1000,H312200,H3y1.00,8CR1.00,9Oi3.00,9Oi4.00,14B4.00,2126200,212G.00,663h.00,1J70.00,1J71.00,663..11,663O.00,663O000,663Q.00,663U.00,663d.00,663,663f.00,663m.00,663n.00,663p.00,663s.00,663y.00,66Y5.00,66Y9.00,66YA.00,66YB.00,66YD.00,66YE.00,66YI.00,66YJ.00,66YK.00,66YL.00,66YL.11,66YM.00,66YQ.00,66YR.00,66YS.00,66YT.00,66YZ.00,66Yd.00,66Ye.00,66Yf.00,66Yh.00,8791,8793,8794,8795,8796,8797,8798,8B3j.00,8CR0.00,8CR1.00,173A.00,173c.00,173d.00,178..00,1780,1O2..00,663N.00,663N000,663N100,663N200,663P.00,663V.00,663V000,663V100,663V200,663V300,663W.00,663e.00,6.63E+102,663j.00,663q.00,663r.00,663t.00,663u.00,663v.00,663w.00,663x.00,66YC.00,66YP.00,66Yg.00,66Yi.00,8H2P.00,8H2R.00,A114.00,A115.00,A789900,AB63400,AD50.00,AD52.00,C350300,C370200,H3...00,H3...11,H30..00,H30..11,H30..12,H300.00,H301.00,H302.00,H30z.00,H31..00,H310.00,H310000,H310100,H310z00,H311.00,H311000,H311100,H311z00,H312.00,H312000,H312011,H312100,H312200,H312300,H312z00,H313.00,H31y.00,H31y000,H31y100,H31yz00,H31z.00,H32..00,H320.00,H320000,H320100,H320200,H320z00,H321.00,H322.00,H32y.00,H32y000,H32y100,H32y111,H32y200,H32yz00,H32z.00,H33..00,H33..11,H330.00,H330.11,H330.12,H330.13,H330.14,H330000,H330011,H330100,H330111,H330z00,H331.00,H331.11,H331000,H331100,H331111,H331z00,H332.00,H333.00,H334.00,H33z.00,H33z.11,H33z000,H33z011,H33z100,H33z111,H33z200,H33zz00,H33zz11,H33zz12,H33zz13,H34..00,H340.00,H341.00,H34z.00,H35..00,H350.00,H351.00,H352.00,H352000,H352100,H352z00,H353.00,H354.00,H355.00,H356.00,H357.00,H35y.00,H35y000,H35y100,H35y200,H35y500,H35y600,H35y700,H35y800,H35yz00,H35z.00,H35z000,H35z100,H35zz00,H36..00,H37..00,H38..00,H39..00,H3y..00,H3y..11,H3y0.00,H3y1.00,H3z..00,H3z..11,H4...00,H4...11,H4...12,H40..00,H41..00,H410.00,H41z.00,H42..00,H420.00,H421.00,H422.00,H423.00,H42z.00,H43..00,H431.00,H432.00,H433.00,H434.00,H435.00,H43z.00,H44..00,H440.00,H441.00,H44z.00,H45..00,H450.00,H460.00,H460100,H460z00,H461.00,H464000,H464100,H464200,H47y000,H48..00,H4y..00,H4y0.00,H4y1.00,H4y1000,H4y1z00,H4z..00,H55..00,H56..00,H560.00,H561.00,H562.00,H563.00,H563.11,H563.12,H563000,H563100,H563z00,H564.00,H56y.00,H56y000,H56y100,H56yz00,H56z.00,H57..00,H570.00,H571.00,H572.00,H57y.00,H57y000,H57y100,H57y200,H57y300,H57y400,H57y500,H57yz00,H58..00,H581.00,H582.00,H58y.00,H58y300,H58yz00,H58z.00,Hyu3000,Hyu3100,Hyu5000,Hyu5100,N042100,N04y000,N04y012 |
| Neurological diseases including:  dementia, Parkinson’s disease, multiple sclerosis | F00,F01,F02,F03,F05, G20,G231,G903,F023,G211,G212,G213,G214,G218,G219,G22 | 3A...12,66h..00,6AB..00,9Ou..00,9Ou1.00,9Ou2.00,9Ou3.00,9Ou4.00,9Ou5.00,9hD..00,9hD0.00,9hD1.00,Eu00.00,Eu00000,Eu00011,Eu00012,Eu00100,Eu00112,Eu00113,Eu00200,Eu00z00,Eu00z11,F110.00,F110000,F110100,Fyu3000,E004.00,E004.11,E004000,E004100,E004200,E004300,E004z00,Eu01.00,Eu01.11,Eu01000,Eu01100,Eu01111,Eu01200,Eu01300,Eu01y00,Eu01z00,E012.00,E012.11,E02y100,E041.00,Eu02.00,Eu02000,Eu02100,Eu02200,Eu02300,Eu02400,Eu02500,Eu02y00,Eu10711,1461,E00..11,E00..12,E000.00,E001.00,E001000,E001100,E001200,E001300,E001z00,E002.00,E002000,E002100,E002z00,E003.00,Eu02z00,Eu02z11,Eu02z13,Eu02z14,Eu02z16,Eu04100,ZS7C500,297A.00,2987,2987.11,2994,2994.11,F116.00,F11x900,F12..00,F12z.00,F130400,F130500,F174.00,F24y000,Eu02300,Eu02500,A94y100,F121.00,F121.11,F123.00,F12W.00,F12X.00,F130300,Fyu2100,Fyu2200,Fyu2900 |
| Depression | F320,F321,F322,F323,F328,F329,F341,F331,F332,F333,F330,F334,F338,F339,F381 | E112100,E112200,Eu32000,Eu32100,Eu32400,Eu32500,Eu32600,E112300,Eu32700,E11..12,E112400,E130.00,E130.11,Eu32311,Eu32312,Eu32313,Eu32314,Eu32800,Eu33311,E112.00,E112000,E112500,E112z00,E11y200,E11z200,E204.00,E2B..00,Eu32.00,Eu32.11,Eu32.12,Eu32.13,Eu32212,Eu32213,Eu32y00,Eu32y11,Eu32y12,Eu32z00,Eu32z11,Eu32z12,Eu32z13,Eu32z14,Eu33z11,Eu34111,Eu34113,E112.12,E112.13,E112.14,Eu33211,E112.11,E135.00,Eu32211,E211200,Eu34100,Eu34112,E2B1.00,E113100,E113200,Eu33000,Eu33100,E113300,Eu33200,E113400,Eu33300,Eu33313,Eu33314,Eu33315,Eu33316,E113.00,E113.11,E113000,E113500,E113600,E113700,E113z00,E118.00,Eu33.00,Eu33.11,Eu33.12,Eu33.13,Eu33.14,Eu33.15,Eu33212,Eu33214,Eu33400,Eu33y00,Eu33z00,Eu3y111,62T1.00,E204.11,E2B0.00,Eu53011,Eu53012,R007z13 |
| Diabetes mellitus | E10,E11,E12,O242,E13,E14,G590,G632,H280,H360,M142,N083,O240,O241,O243 | C104.00,C104.11,C104000,C104100,C104y00,C104z00,C108000,C108011,C108012,C108D00,C108D11,C109000,C109011,C109012,C109C00,C109C11,C109C12,C10E000,C10ED00,C10EK00,C10EL00,C10F000,C10F011,C10FC00,C10FL00,C10FL11,C10FM00,C10FM11,C314.11,K01x100,K01x111,C106.00,C106.11,C106.12,C106.13,C106000,C106100,C106y00,C106z00,C108200,C108211,C108212,C108B00,C108C00,C108J00,C108J11,C108J12,C109200,C109211,C109212,C109A00,C109A11,C109B00,C109B11,C109H00,C109H11,C109H12,C10E200,C10EB00,C10EC00,C10EC11,C10EJ00,C10EQ00,C10F200,C10F211,C10FA00,C10FA11,C10FB00,C10FB11,C10FH00,C10FR00,F171100,F345000,F35z000,F372.00,F372.11,F372.12,F372000,F372100,F372200,F381300,F381311,F3y0.00,M271100,N030100,C105.00,C105000,C105100,C105y00,C105z00,C108100,C108F00,C108F11,C109100,C109111,C109112,C109E00,C109E11,C109E12,C10E100,C10E112,C10EF00,C10F100,C10FE00,C10FE11,F464000,2BBF.00,C108700,C108711,C108712,C109600,C109611,C109612,C10E700,C10E711,C10E712,C10F600,C10F611,F420.00,F420z00,2BBP.00,2BBQ.00,F420000,2BBR.00,2BBS.00,F420200,F420500,F420600,F420800,2BBT.00,2BBV.00,2BBo.00,F420100,F420700,2BBk.00,2BBl.00,7276,2BBL.00,2BBW.00,2BBX.00,C10EP00,C10EP11,C10FQ00,F420300,F420400,1434,14F4.00,14P3.00,2126300,212H.00,9OL9.00,13Y1.00,3881,3882,66A..00,66A1.00,66A2.00,66AM.00,66AZ.00,66Af.00,66Ak.00,66Al.00,679L.00,679R.00,8A12.00,8A17.00,8A18.00,8A19.00,8A1A.00,8CR2.00,8CS0.00,8HHy.00,8HTe.00,8HTk.00,8Hg4.00,8Hj0.00,8Hj3.00,8Hj4.00,8Hj5.00,8I6F.00,8I6G.00,8I81.00,8I82.00,8I83.00,8I84.00,93C4.00,9N0m.00,9N0n.00,9N0o.00,9N1Q.00,9N1i.00,9N1o.00,9N1v.00,9N2d.00,9N2i.00,9N4I.00,9N4p.00,9NM0.00,9NN8.00,9NN9.00,9NND.00,9NiA.00,9NiD.00,9NiE.00,9Nl4.00,9OL..00,9OL1.00,9OL2.00,9OL3.00,9OL4.00,9OL5.00,9OL6.00,9OL7.00,9OL8.00,9OLA.00,9OLA.11,9OLB.00,9OLF.00,9OLG.00,9OLH.00,9OLJ.00,9OLK.00,9OLL.00,9OLM.00,9OLZ.00,9h4..00,9h41.00,9h42.00,ZL22500,ZLA2500,ZLD7500,ZRB4.00,ZRB4.11,ZRB5.00,ZRB5.11,ZRB6.00,ZRB6.11,ZRBa.00,66An.00,C100000,C100011,C101000,C102000,C103000,C104000,C105000,C106000,C107000,C107300,C108.00,C108.11,C108.12,C108.13,C108000,C108011,C108012,C108100,C108200,C108211,C108212,C108300,C108400,C108411,C108412,C108500,C108511,C108512,C108600,C108700,C108711,C108712,C108800,C108811,C108812,C108900,C108911,C108912,C108A00,C108A11,C108B00,C108C00,C108D00,C108D11,C108E00,C108E11,C108E12,C108F00,C108F11,C108G00,C108H00,C108H11,C108J00,C108J11,C108J12,C10C.12,C10E.00,C10E.11,C10E.12,C10E000,C10E100,C10E112,C10E200,C10E300,C10E311,C10E312,C10E400,C10E411,C10E412,C10E500,C10E511,C10E512,C10E600,C10E700,C10E711,C10E712,C10E800,C10E812,C10E900,C10E911,C10E912,C10EA00,C10EA11,C10EB00,C10EC00,C10EC11,C10ED00,C10EE00,C10EF00,C10EG00,C10EH00,C10EJ00,C10EK00,C10EL00,C10EM00,C10EM11,C10EN00,C10EN11,C10EP00,C10EP11,C10EQ00,C10z000,L180500,M21yC00,M21yC11,ZC2C900,ZRbH.00,66Ao.00,C100100,C100111,C100112,C101100,C102100,C103100,C104100,C105100,C106100,C107100,C107200,C107400,C109.00,C109.11,C109.12,C109.13,C109000,C109011,C109012,C109100,C109111,C109112,C109200,C109211,C109212,C109300,C109400,C109411,C109412,C109500,C109511,C109512,C109600,C109611,C109612,C109700,C109711,C109712,C109900,C109A00,C109A11,C109B00,C109B11,C109C00,C109C11,C109C12,C109D00,C109D11,C109D12,C109E00,C109E11,C109E12,C109F00,C109F11,C109F12,C109G00,C109G11,C109G12,C109H00,C109H11,C109H12,C109J00,C109J11,C109J12,C109K00,C10C.11,C10D.00,C10D.11,C10ER00,C10F.00,C10F.11,C10F000,C10F011,C10F100,C10F200,C10F211,C10F300,C10F311,C10F400,C10F411,C10F500,C10F600,C10F611,C10F700,C10F711,C10F900,C10F911,C10FA00,C10FA11,C10FB00,C10FB11,C10FC00,C10FD00,C10FD11,C10FE00,C10FE11,C10FF00,C10FG00,C10FH00,C10FJ00,C10FJ11,C10FK00,C10FL00,C10FL11,C10FM00,C10FM11,C10FN00,C10FP00,C10FQ00,C10FR00,C10y100,C10z100,L180600,ZC2CA00,C10B.00,C10B000,C10FS00,C10G.00,C10G000,C10H.00,C10H000,C10N.00,C10N000,C10N100,C11y000,13AB.00,13AC.00,13B1.00,2BBF.00,2BBL.00,2BBM.00,2BBP.00,2BBQ.00,2BBR.00,2BBS.00,2BBT.00,2BBV.00,2BBW.00,2BBX.00,2BBk.00,2BBl.00,2BBo.00,2G51000,2G5A.00,2G5B.00,2G5C.00,2G5E.00,2G5F.00,2G5G.00,2G5H.00,2G5I.00,2G5J.00,2G5K.00,2G5L.00,2G5V.00,2G5W.00,66A3.00,66A4.00,66A5.00,66A8.00,66A9.00,66AA.11,66AD.00,66AG.00,66AH.00,66AI.00,66AJ.00,66AJ.11,66AJ100,66AJz00,66AK.00,66AL.00,66AN.00,66AO.00,66AP.00,66AQ.00,66AR.00,66AS.00,66AT.00,66AU.00,66AV.00,66AW.00,66AX.00,66AY.00,66Aa.00,66Ab.00,66Ac.00,66Ag.00,66Ah.00,66Ai.00,66Aj.00,66Am.00,66Ap.00,66Aq.00,6761,68A7.00,68A9.00,68AB.00,7276,7L10000,7L19800,889A.00,8A13.00,8B3l.00,8BL2.00,8CA4100,8CAQ.00,8CP2.00,8H2J.00,8H3O.00,8H7r.00,8HBG.00,8HBH.00,8HLE.00,8Hl1.00,8I3W.00,8I3X.00,8I3k.00,8I57.00,9360,9OLD.00,C10..00,C100.00,C100z00,C101.00,C101y00,C101z00,C102.00,C102z00,C103.00,C103y00,C103z00,C104.00,C104.11,C104y00,C104z00,C105.00,C105y00,C105z00,C106.00,C106.11,C106.12,C106.13,C106y00,C106z00,C107.00,C107.11,C107.12,C107z00,C108y00,C108z00,C10A.00,C10A000,C10A100,C10C.00,C10M.00,C10y.00,C10yy00,C10yz00,C10z.00,C10zy00,C10zz00,C314.11,C350011,Cyu2.00,Cyu2000,F171100,F345000,F35z000,F372.00,F372.11,F372.12,F372000,F372100,F372200,F381300,F381311,F3y0.00,F420.00,F420000,F420100,F420200,F420300,F420400,F420500,F420600,F420700,F420800,F420z00,F440700,F464000,G73y000,K01x100,K01x111,L180X00,M037200,M271000,M271100,M271200,N030000,N030011,N030100,Q441.00,R054200,R054300,TJ23.00,TJ23z00,U602311,ZC2C800,ZV65312,1I0..00 |
| Inflammatory diseases including: inflammatory bowel disease, psoriasis, lupus erythematosus, osteoarthritis, connective tissue diseases, Sjogren’s syndrom | G35X,K510,K512,K513,K515,K518,K519,M075,K50,M074,L400,L401,L402,L403,L404,L405,L408,L409,M070,M071,M072,M073,M090,M320,M321,M328,M329,M15,M16,M17,M18,M19,M471,M472,M478,M479,M05,M060,M063,M069,M32,M332,M34,M353,M330,M331,M339,M350 | F20..00,F203.00,F20..11,F20z.00,F202.00,F200.00,666B.00,666A.00,8CS1.00,F201.00,14C4.11,J41..00,J41..12,J410.00,J410000,J410100,J410300,J410400,J410z00,J411.00,J412.00,J41y.00,J41yz00,J41z.00,Jyu4100,N031000,N045400,J08z900,J40..00,J40..11,J400200,J400300,J400400,J400500,J400z00,J401200,J401z00,J401z11,J40z.11,Jyu4000,N031100,N045300,ZR3S.00,ZR3S.11,J4...12,14F2.00,M160000,M161.00,M161000,M161100,M161200,M161300,M161400,M161500,M161600,M161700,M161800,M161900,M161A00,M161B00,M161C00,M161D00,M161E00,M161F00,M161F11,M161H00,M161z00,M16y.00,M16y000,Myu3000,N045200,M160.00,M160100,M160.11,M160z00,Nyu1300,M160200,12H2.00,12H5.00,M16..00,M162.00,M162000,M162100,M162300,M162400,M162500,M162600,M162700,M162900,M162A00,M162z00,M16z.00,Myu3100,M154.00,M154z00,N000.00,N000400,N000000,K01x411,M154700,N000300,H57y400,N000200,K0B4000,N000z00,F371000,ZRq9.00,K01x400,ZRq8.00,N000100,Nyu4300,ZRq9.11,12I2.00,7L0H100,7L0H112,N040000,N040100,N090.11,N090W00,N091.00,N091000,N091100,N091200,N091211,N091300,N091400,N091500,N091511,N091600,N091611,N091700,N091711,N091800,N091900,N091A00,N091D00,N091F00,N091H00,N091K00,N091M00,N091N00,N091P00,N091Q00,N091S00,N091V00,N091z00,N093.12,N338100,N33zC00,PF6y600,14G2.00,2G26.00,N05..00,N05..11,N050.00,N050000,N050100,N050111,N050112,N050200,N050300,N050400,N050500,N050600,N050700,N050z00,N051.00,N051000,N051100,N051200,N051300,N051400,N051500,N051600,N051700,N051800,N051900,N051A00,N051B00,N051C00,N051D00,N051E00,N051F00,N051z00,N052.00,N052000,N052100,N052200,N052300,N052400,N052500,N052600,N052700,N052800,N052900,N052A00,N052B00,N052C00,N052z00,N053.00,N053000,N053100,N053200,N053300,N053400,N053500,N053512,N053600,N053611,N053700,N053800,N053900,N053z00,N054.00,N054000,N054100,N054200,N054400,N054500,N054600,N054700,N054800,N054900,N054z00,N05z.00,N05z.11,N05z000,N05z100,N05z200,N05z211,N05z300,N05z311,N05z400,N05z411,N05z412,N05z500,N05z511,N05z600,N05z611,N05z700,N05z711,N05z712,N05z713,N05z800,N05z900,N05zA00,N05zB00,N05zC00,N05zD00,N05zE00,N05zF00,N05zG00,N05zH00,N05zJ00,N05zK00,N05zL00,N05zM00,N05zN00,N05zP00,N05zQ00,N05zR00,N05zS00,N05zT00,N05zU00,N05zz00,Nyu2.00,Nyu2000,Nyu2100,Nyu2400,Nyu2500,Nyu2511,Nyu2700,Nyu2800,Nyu2811,Nyu2D00,Nyu2E11,Nyu2F00,F163200,F337200,N11..00,N11..11,N11..12,N110.00,N110.11,N110.12,N110000,N110100,N110200,N111.00,N111000,N111100,N111200,N112.00,N112.11,N112000,N112100,N112200,N112300,N113.00,N113000,N113200,N114.00,N114.11,N114.12,N114000,N114100,N114200,N115.00,N115000,N115100,N115200,N119.00,N119000,N119100,N119200,N11A.00,N11B.00,N11B000,N11B200,N11C.00,N11C000,N11C100,N11C200,N11D.00,N11D000,N11D100,N11D200,N11D300,N11E.00,N11z.00,N11z.11,N11z000,N11z100,N11zz00,Nyu6300,Nyu6400,14G1.00,66H..13,F371000,F371200,F396400,F396600,F4D3300,G5y8.00,G5yA.00,H570.00,H572.00,H57y100,H57y400,K01x400,K01x411,M154.00,M154000,M154100,M154200,M154300,M154400,M154500,M154600,M154700,M154z00,M210.00,M210000,M210400,M210z00,N000.00,N000000,N000100,N000200,N000300,N000400,N000z00,N001.00,N001.11,N001.12,N001000,N001100,N004.00,N04..00,N040.00,N040000,N040100,N040200,N040500,N040600,N040700,N040800,N040900,N040A00,N040B00,N040D00,N040F00,N040G00,N040H00,N040J00,N040K00,N040N00,N040P00,N040R00,N040S00,N040T00,N041.00,N042.00,N042100,N042200,N042z00,N043.00,N043000,N043100,N043200,N043300,N043z00,N044.00,N045500,N047.00,N04X.00,N04y000,N04y011,N04y012,N20..00,N20..11,N200.00,N231400,Nyu1100,Nyu1200,Nyu1G00,Nyu4300,Nyu4500,Nyu4F00,N003.00,N003.11,N003000,N003100,N003X00,Nyu4400,Nyu4E00,N002.11,N002.00,F396700,H57y300 |
| Liver diseases | K702,K703,K717,K73,K740,K742,K743,K744,K745,K746,I85,K721,K729,K766,K767 | C310400,C350012,J61..00,J612.00,J612.11,J614.00,J614000,J614100,J614200,J614400,J614y00,J614z00,J615.00,J615.11,J615100,J615300,J615400,J615500,J615600,J615700,J615800,J615812,J615D00,J615H00,J615y00,J615z00,J615z11,J615z12,J615z13,J616.00,J616000,J616100,J616200,J616z00,J617000,J61y300,J635600,Jyu7100,7609,7609300,7609400,7609z00,760C500,760F300,760F400,G85..11,G850.00,G851.00,G852.00,G852000,G852100,G852200,G852300,G852z00,G858.00,J622.00,J622.11,J623.00,J624.00,J625.00,J625.11,J62y.11,J62y.12,J62y.13 |
| Renal diseases | N181,N182,N00,N10,N17,N01,N03,N052,N053,N054,N055,N056,N072,N073,N074,N183,N184,N185,N189,N19,N25,Y841,Z49,Z992,T861,Z940 | 14D..11,14D..12,14D1.00,14V2.00,14V2.11,1Z10.00,1Z11.00,1Z17.00,1Z18.00,1Z19.00,1Z1A.00,A844100,K00..00,K00..11,K000.00,K001.00,K00y.00,K00y000,K00y100,K00y200,K00y300,K00yz00,K00z.00,K03T.00,K04..00,K040.00,K041.00,K042.00,K043.00,K044.00,K04y.00,K04z.00,K08y500,K0A0.00,K0A0100,K0A0200,K0A0500,K0A0700,K101.00,K101000,K101z00,Kyu2000,L393.00,L393000,L393100,L393200,SK05.00,SK05.11,SK08.00,SP15412,1Z1..00,1Z12.00,1Z13.00,1Z14.00,1Z15.00,1Z16.00,1Z1B.00,1Z1C.00,1Z1D.00,1Z1E.00,1Z1F.00,1Z1G.00,1Z1H.00,1Z1J.00,1Z1K.00,1Z1L.00,7A60600,7B0F100,7L1A.00,7L1Ay00,7L1Az00,7L1B.00,7L1By00,7L1C.00,7L1Cz00,8L50.00,A160000,A160200,A786.00,C104.11,C104z00,C108D00,C108D11,C109C00,C109C11,C109C12,C10ED00,C10FC00,C341.00,C341z00,C345.00,C373600,D111300,D215.00,D215000,D310100,F374A00,G22..00,G220.00,G221.00,G222.00,G22z.00,G23..00,G230.00,G231.00,G232.00,G233.00,G23z.00,G500400,Gyu2100,K0...00,K01..00,K010.00,K011.00,K013.00,K013.12,K014.00,K015.00,K016.00,K017.00,K018.00,K019.00,K01A.00,K01B.00,K01w.00,K01x000,K01x100,K01x300,K01x400,K01x411,K01y.00,K01z.00,K02..00,K02..11,K02..12,K020.00,K021.00,K022.00,K023.00,K02y.00,K02y000,K02y200,K02y300,K02yz00,K02z.00,K03..00,K03..11,K03..12,K030.00,K031.00,K032.00,K032000,K032300,K032400,K032500,K032600,K032y00,K032y11,K032y13,K032y14,K032y15,K032z00,K033.00,K034.00,K035.00,K03U.00,K03V.00,K03W.00,K03X.00,K03y.00,K03y000,K03y200,K03yz00,K03z.00,K05..00,K05..11,K05..12,K050.00,K06..00,K06..11,K060.00,K060.11,K08..00,K080.00,K080000,K080100,K080200,K080300,K080z00,K081.00,K08y.00,K08y000,K08y300,K08y400,K08yz00,K08yz11,K08z.00,K0A0300,K0A1.00,K0A1100,K0A1200,K0A1300,K0A1600,K0A1700,K0A2200,K0A2300,K0A2500,K0A2700,K0A2800,K0A3.00,K0A3000,K0A3100,K0A3200,K0A3300,K0A3500,K0A3600,K0A3700,K0A4500,K0A5.00,K0A5000,K0A5100,K0A5200,K0A5300,K0A5600,K0A5X00,K0B..00,K0B1.00,K0B4000,K0C0.00,K0C1.00,K0C2.00,K0C4.00,K0D..00,K0y..00,K0z..00,K100.00,K100100,K100400,K100500,K100600,K100z00,K104.00,K10y.00,K10y000,K10y300,K10yz00,K13..00,K13..11,K138.00,K138.11,K138z00,K13y.00,K13yz00,K13yz11,K13z.00,Kyu0900,Kyu1.00,Kyu1400,Kyu2.00,Kyu2100,Kyu4.00,Kyu4000,Kyu4100,L093.00,L093400,L162.00,L162.12,L162.13,L162000,L162100,PD1..00,PD1..11,PD11.00,PD11z00,PD11z11,PD1y.00,PD1y000,PD1yz00,PD1z.00,Pyu7000,Q001.00,Q48y000,SP15400,SP15411,SP15413,Z1A2.00,ZV56100,7A61900,7L1A.11,7L1A000,7L1A100,7L1A200,7L1A400,7L1A500,7L1A600,7L1B.11,7L1B000,7L1B100,7L1C000,SP01500,SP05613,SP07G00,TA02000,TA22000,TB11.00,TB11.11,U612200,Z919.00,Z919100,Z919300,Z91A.00,ZV45100,ZV56.00,ZV56011,ZV56y00,ZV56y11,ZV56z00,ZVu3G00,7B00.00,7B00000,7B00100,7B00111,7B00200,7B00211,7B00300,7B00400,7B00y00,7B00z00,7B01500,7B01511,7B06300,7B0F.00,K0B5.00,SP08300,TB00100,TB00111,ZV42000,66i..00,6AA..00,9Ot..00,9Ot0.00,9Ot1.00,9Ot2.00,9Ot3.00,9Ot4.00,, |

**Key:** International Classification of Diseases 10^th^ Edition (ICD-10), version 2 (v2).
