# Supplementary material for: The additive effect of periodontitis with hypertension on risk of systemic disease and mortality
Source: J Periodontol. 2022 May 27;93(7):1024–35. doi: 10.1002/JPER.21-0621 (PMC9544472; doi:10.1002/JPER.21-0621)
Supplement: Supplementary file 2 — Supplemental Table 2. Antihypertensive medications. [file JPER-93-1024-s004.docx]

**Supplemental Table 2** Antihypertensive medications.

| **Drug Substance** | **Product Names** | **Read (v2) codes** | **DM+D codes** | **BNF codes** |
| --- | --- | --- | --- | --- |
| Sacubitril, Valsartan, Methyldopa anhydrous, Guanfacine hydrochloride, Moxonidine, Clonidine hydrochloride, Alseroxylon, Clonidine, Reserpine, Theobromine, Phenobarbital, Benzthiazide, Methoserpidine, Bendroflumethiazide, Hydrochlorothiazide, Methyldopa Anhydrous, Methyldopate Hydrochloride, Amobarbital Sodium, Rauwolfia, Iloprost trometamol, Hydralazine hydrochloride, Riociguat, Minoxidil, Ambrisentan, Sitaxentan sodium, Sildenafil citrate, Selexipag, Bosentan monohydrate, Tadalafil, Sodium nitroprusside dihydrate, Macitentan, Hydralazine Hydrochloride, Sildenafil Citrate | Sacubitril, Entresto, RESPeRATE, Dopamet, Guanfacine, Moxonidine, Clonidine, Rauwiloid, Hypercal, Methyldopa, Physiotens, Serpasil, Seominal, Decaserpyl, Abicol, Aldomet, Methoserpidine, Catapres, Metalpha, Reserpine, Hydromet, Intuniv, Iloprost, Hydralazine, Adempas, Loniten, Ambrisentan, Sitaxentan, Revatio, Apresoline, Selexipag, Riociguat, Minoxidil, Bosentan, Sildenafil, Thelin, Adcirca, Ventavis, Tracleer, Sodium, Macitentan, Regaine, Tadalafil | 66931, 66702, 66197, 66829, 66261, 66205, 49290, 24196, 67005, 43531, 6694, 2104, 23345, 3070, 68529, 49684, 9876, 32913, 60898, 20656, 25645, 11177, 4406, 30129, 28738, 71110, 10714, 62853, 19892, 62513, 43988, 25393, 16248, 7642, 8033, 29187, 1707, 61036, 78159, 76766, 25275, 29570, 15493, 2878, 23380, 7174, 67516, 9225, 10253, 29696, 41661, 21346, 43989, 54467, 4215, 72819, 7416, 7626, 73640, 76479, 30293, 21502, 67665, 26919, 78157, 67675, 4993, 14390, 30691, 78162, 66936, 22853, 10713, 67808, 20690, 66388, 25289, 77825, 33322, 63938, 58529, 66429, 23761, 40310, 3049, 71385, 9749, 66016, 18252, 72807, 60136, 53142, 61256, 61710, 67200, 52555, 77591, 55797, 74534, 58090, 72130, 51328, 18861, 74720, 9463, 40528, 37085, 46249, 40527, 504, 53896, 2680, 36840, 76516, 75040, 2968, 29560, 2967, 27137, 14495, 40899, 74749, 31220, 46795, 61116, 47264, 9697, 30967, 1296, 76902, 2362, 41639, 75259, 71256, 64930, 2970, 38519, 70655, 58632, 75041, 36612, 64253, 573, 43500, 47654, 63780, 55368, 63578, 45641, 13317, 59512, 71097, 29561, 63652, 24797, 35693, 66468, 46055, 35116, 70126, 58151, 62659, 49411, 70587, 69454, 50362, 35192, 59771, 6207, 72637 | 31142211000001100, 31142011000001100, 31142111000001100, 31138011000001100, 31136811000001100, 31136411000001100, 19880911000001100, 54625001000027104, 31087611000001100, 8099411000001100, 318668000, 26725001000027100, 139675001000027008, 318673006, 31087811000001100, 10448511000001100, 522011000001109, 874511000001106, 8171411000001100, 76555001000027104, 49555001000027104, 41111000001102, 48465001000027104, 45001000027103, 125055001000027008, 17895811000001100, 124465001000027008, 8936111000001100, 76575001000027104, 17204211000001100, 253711000001107, 6865001000027100, 36089211000001104, 665001000027102, 645001000027108, 124475001000027008, 318672001, 21407711000001100, 42925001000027104, 10449311000001100, 29705001000027100, 54615001000027104, 139855001000027008, 318667005, 368711000001103, 318708005, 31087711000001100, 204605001000027008, 408604009, 124515001000027008, 15965001000027100, 125115001000027008, 73611000001108, 10449111000001100, 215111000001101, 17940911000001100, 51165001000027104, 655001000027106, 87911000001103, 8938711000001100, 364911000001108, 189705001000027008, 8265311000001100, 125025001000027008, 15955001000027100, 31087911000001100, 318707000, 51145001000027104, 181845001000027008, 88511000001109, 31074311000001100, 139805001000027008, 6855001000027100, 21406211000001100, 139815001000027008, 31074611000001100, 54635001000027104, 233711000001108, 8098911000001100, 8099111000001100, 7660211000001100, 31074911000001100, 8667311000001100, 8390411000001100, 318671008, 17895611000001100, 142811000001107, 31074011000001100, 29695001000027100, 11813311000001100, 11812911000001100, 8398611000001100, 8426311000001100, 18402811000001100, 8397911000001100, 8398511000001100, 8398211000001100, 8398311000001100, 32392011000001100, 11814111000001100, 11813011000001100, 4897711000001100, 8528611000001100, 24399711000001100, 3666411000001100, 429662009, 11394311000001100, 18759511000001100, 428480001, 34193811000001100, 36046411000001104, 1655001000027100, 420370000, 32963211000001100, 24408411000001100, 318657000, 407815004, 318656009, 419015002, 3666711000001100, 11392011000001100, 414426001, 193711000001103, 18759411000001100, 8528811000001100, 18249011000001100, 3667011000001100, 10154511000001100, 318650003, 24408711000001100, 657011000001101, 147111000001103, 12536611000001100, 30172911000001100, 24408611000001100, 318655008, 11383311000001100, 8581011000001100, 4765711000001100, 24408311000001100, 375899001, 12537811000001100, 318649003, 364711000001106, 4765211000001100, 23707811000001100, 21705011000001100, 21700211000001100, 16660911000001100, 3925011000001100, 8528711000001100, 19819011000001100, 407816003, 245915001000027008, 9524811000001100, 11465411000001100, 32066411000001100, 18550611000001100, 11472511000001100, 14160211000001100, 15452411000001100, 15535711000001100, 15452511000001100, 14058011000001100, 15650211000001100, 15535611000001100, 246975001000027008, 14058111000001100, 407858000, 34912511000001100 | 02050502, 02050600, 2050700, 2050200, 02050200, 04070402, 06040101, 2050100, 02050100, 13090300, 07040500, 07040501 |

**Key:** British National Formulary (BNF), Dictionary of medicines and devices(DM+D), International Classification of Diseases 10^th^ Edition (ICD-10), version 2 (v2)
