# Supplementary material for: The additive effect of periodontitis with hypertension on risk of systemic disease and mortality
Source: J Periodontol. 2022 May 27;93(7):1024–35. doi: 10.1002/JPER.21-0621 (PMC9544472; doi:10.1002/JPER.21-0621)
Supplement: Supplementary file 3 — Supplemental Table 3. Association between oral health indicators and risk of subsequent systemic disease compared to healthy controls, stratified by hypertension category and using complete cases only. [file JPER-93-1024-s001.docx]

**Supplemental Table 3.** Association between oral health indicators and risk of subsequent systemic disease compared to healthy controls, stratified by hypertension category and using complete cases only.

|  |  | **Non-hypertensive** | | | **Hypertensive** | | |
| --- | --- | --- | --- | --- | --- | --- | --- |
| **Systemic  disease/all-cause mortality** | **Hazard Ratio (95%CI)** | **Healthy** | **Painful gums** | **Loose teeth** | **Healthy** | **Painful gums** | **Loose teeth** |
| **Cancer** | Crude | **1 (ref)** | 1.03 (0.92 - 1.15) | 1.18 (1.06 - 1.31) | 1.36 (1.33 - 1.39) | 1.20 (1.08 - 1.33) | 1.43 (1.31 - 1.55) |
|  | Adjusted | **1 (ref)** | 1.03 (0.89 - 1.19) | 1.03 (0.90 - 1.17) | 1.00 (0.97 - 1.03) | 0.97 (0.84 - 1.11) | 0.96 (0.86 - 1.06) |
| **CVD** | Crude | **1 (ref)** | 1.22 (1.07 - 1.40) | 1.46 (1.29 - 1.66) | 2.03 (1.98 - 2.09) | 2.43 (2.20 - 2.68) | 2.60 (2.40 - 2.82) |
|  | Adjusted | **1 (ref)** | 1.23 (1.03 - 1.46) | 1.23 (1.05 - 1.43) | 1.31 (1.26 - 1.36) | 1.58 (1.38 - 1.80) | 1.51 (1.36 - 1.67) |
| **Diabetes** | Crude | **1 (ref)** | 1.08 (0.93 - 1.25) | 0.93 (0.79 - 1.09) | 0.89 (0.86 - 0.92) | 0.93 (0.79 - 1.08) | 0.69 (0.61 - 0.78) |
|  | Adjusted | **1 (ref)** | 1.05 (0.86 - 1.28) | 1.09 (0.89 - 1.34) | 0.96 (0.91 - 1.01) | 1.01 (0.82 - 1.24) | 0.79 (0.67 - 0.93) |
| **Depression** | Crude | **1 (ref)** | 1.41 (1.14 - 1.76) | 1.45 (1.17 - 1.80) | 1.03 (0.98 - 1.08) | 1.58 (1.29 - 1.94) | 1.16 (0.95 - 1.42) |
|  | Adjusted | **1 (ref)** | 1.21 (0.91 - 1.61) | 1.48 (1.16 - 1.90) | 0.97 (0.90 - 1.05) | 1.48 (1.13 - 1.93) | 0.95 (0.74 - 1.23) |
| **Inflammatory disease** | Crude | **1 (ref)** | 1.12 (1.00 - 1.25) | 1.19 (1.06 - 1.32) | 1.44 (1.41 - 1.47) | 1.50 (1.36 - 1.65) | 1.61 (1.49 - 1.75) |
|  | Adjusted | **1 (ref)** | 1.05 (0.91 - 1.22) | 1.01 (0.88 - 1.16) | 1.01 (0.98 - 1.04) | 1.00 (0.88 - 1.15) | 1.00 (0.91 - 1.11) |
| **Liver disease** | Crude | **1 (ref)** | 1.56 (0.85 - 2.84) | 1.96 (1.15 - 3.35) | 1.88 (1.65 - 2.14) | 2.82 (1.79 - 4.42) | 2.60 (1.76 - 3.85) |
|  | Adjusted | **1 (ref)** | 1.49 (0.70 - 3.17) | 1.75 (0.95 - 3.22) | 1.25 (1.04 - 1.52) | 1.58 (0.85 - 2.93) | 1.16 (0.69 - 1.96) |
| **Neurological disease** | Crude | **1 (ref)** | 0.86 (0.51 - 1.43) | 1.42 (0.95 - 2.11) | 1.89 (1.74 - 2.05) | 1.65 (1.14 - 2.39) | 2.11 (1.60 - 2.78) |
|  | Adjusted | **1 (ref)** | 0.98 (0.51 - 1.91) | 1.08 (0.66 - 1.78) | 0.99 (0.87 - 1.12) | 0.91 (0.52 - 1.58) | 0.85 (0.58 - 1.25) |
| **Renal disease** | Crude | **1 (ref)** | 1.34 (1.07 - 1.69) | 1.70 (1.39 - 2.09) | 2.51 (2.40 - 2.62) | 2.91 (2.48 - 3.41) | 3.21 (2.82 - 3.64) |
|  | Adjusted | **1 (ref)** | 1.31 (0.97 - 1.77) | 1.43 (1.12 - 1.83) | 1.41 (1.32 - 1.51) | 1.52 (1.22 - 1.90) | 1.47 (1.25 - 1.73) |
| **Respiratory disease** | Crude | **1 (ref)** | 1.33 (1.12 - 1.57) | 1.57 (1.35 - 1.83) | 1.31 (1.27 - 1.36) | 1.81 (1.57 - 2.09) | 2.07 (1.85 - 2.31) |
|  | Adjusted | **1 (ref)** | 1.24 (1.00 - 1.53) | 1.40 (1.17 - 1.68) | 1.04 (0.99 - 1.10) | 1.34 (1.11 - 1.62) | 1.41 (1.23 - 1.62) |
| **All-cause mortality** | Crude | **1 (ref)** | 1.25 (1.01 - 1.54) | 1.89 (1.59 - 2.24) | 1.79 (1.72 - 1.87) | 1.97 (1.66 - 2.34) | 2.95 (2.62 - 3.32) |
|  | Adjusted | **1 (ref)** | 1.02 (0.78 - 1.32) | 0.96 (0.77 - 1.18) | 1.03 (0.97 - 1.09) | 1.03 (0.82 - 1.30) | 0.93 (0.80 - 1.09) |

**Key:** body mass index (BMI), confidence interval (CI), cardiovascular disease (CVD), hazard ratio (HR), periodontitis (PD, reference value (ref), insufficient numbers(-)
***** Adjusted by age, sex, BMI, ethnicity, average total household income, C-reactive protein level, history of smoking, time since hypertension diagnosis, death and hypertension*PD interaction
