# Supplementary material for: The additive effect of periodontitis with hypertension on risk of systemic disease and mortality
Source: J Periodontol. 2022 May 27;93(7):1024–35. doi: 10.1002/JPER.21-0621 (PMC9544472; doi:10.1002/JPER.21-0621)
Supplement: Supplementary file 4 — Supplemental Table 4. Association between oral health indicators and risk of subsequent systemic disease compared to healthy controls and excluding those on antihypertensive medication, stratified by hypertension category. [file JPER-93-1024-s003.docx]

**Supplemental Table 4.** Association between oral health indicators and risk of subsequent systemic disease compared to healthy controls and excluding those on antihypertensive medication, stratified by hypertension category.

|  |  | **Non-hypertensive** | | | **Hypertensive** | | |
| --- | --- | --- | --- | --- | --- | --- | --- |
| **Systemic  disease/all-cause mortality** | **Hazard Ratio (95%CI)** | **Healthy** | **Painful gums** | **Loose teeth** | **Healthy** | **Painful gums** | **Loose teeth** |
| **Cancer** | Crude | **1 (ref)** | 1.01 (0.89 - 1.14) | 1.20 (1.07 - 1.35) | 1.36 (1.33 - 1.39) | 1.15 (1.03 - 1.30) | 1.42 (1.30 - 1.55) |
|  | Adjusted | **1 (ref)** | 1.03 (0.91 - 1.16) | 1.01 (0.90 - 1.13) | 1.01 (0.98 - 1.03) | 0.89 (0.79 - 1.00) | 0.92 (0.85 - 1.01) |
| **CVD** | Crude | **1 (ref)** | 1.22 (1.06 - 1.41) | 1.50 (1.31 - 1.71) | 2.05 (2.00 - 2.11) | 2.38 (2.13 - 2.65) | 2.59 (2.38 - 2.83) |
|  | Adjusted | **1 (ref)** | 1.26 (1.09 - 1.46) | 1.19 (1.04 - 1.35) | 1.30 (1.26 - 1.34) | 1.60 (1.44 - 1.79) | 1.40 (1.29 - 1.53) |
| **Diabetes** | Crude | **1 (ref)** | 1.56 (1.35 - 1.81) | 1.42 (1.22 - 1.65) | 1.52 (1.48 - 1.58) | 2.00 (1.75 - 2.27) | 2.01 (1.81 - 2.24) |
|  | Adjusted | **1 (ref)** | 1.44 (1.25 - 1.67) | 1.16 (1.00 - 1.35) | 1.20 (1.16 - 1.24) | 1.48 (1.30 - 1.68) | 1.35 (1.21 - 1.51) |
| **Depression** | Crude | **1 (ref)** | 1.32 (1.04 - 1.68) | 1.40 (1.11 - 1.76) | 1.02 (0.97 - 1.08) | 1.61 (1.30 - 2.01) | 1.12 (0.90 - 1.38) |
|  | Adjusted | **1 (ref)** | 1.22 (0.96 - 1.55) | 1.28 (1.02 - 1.62) | 0.96 (0.90 - 1.02) | 1.37 (1.10 - 1.71) | 0.94 (0.76 - 1.17) |
| **Inflammatory disease** | Crude | **1 (ref)** | 1.14 (1.01 - 1.28) | 1.17 (1.04 - 1.31) | 1.45 (1.42 - 1.48) | 1.55 (1.40 - 1.72) | 1.57 (1.45 - 1.71) |
|  | Adjusted | **1 (ref)** | 1.11 (0.98 - 1.25) | 0.98 (0.87 - 1.10) | 1.00 (0.97 - 1.02) | 1.07 (0.96 - 1.19) | 0.97 (0.89 - 1.05) |
| **Liver disease** | Crude | **1 (ref)** | 1.10 (0.52 - 2.34) | 1.87 (1.05 - 3.33) | 1.90 (1.65 - 2.18) | 2.59 (1.56 - 4.28) | 2.50 (1.64 - 3.82) |
|  | Adjusted | **1 (ref)** | 1.04 (0.49 - 2.21) | 1.40 (0.79 - 2.50) | 1.28 (1.10 - 1.49) | 1.59 (0.95 - 2.64) | 1.27 (0.82 - 1.95) |
| **Neurological disease** | Crude | **1 (ref)** | 0.81 (0.47 - 1.41) | 1.36 (0.89 - 2.08) | 1.86 (1.71 - 2.03) | 1.61 (1.08 - 2.39) | 2.04 (1.52 - 2.73) |
|  | Adjusted | **1 (ref)** | 0.80 (0.46 - 1.39) | 0.90 (0.59 - 1.38) | 0.99 (0.90 - 1.09) | 0.94 (0.63 - 1.40) | 0.85 (0.63 - 1.15) |
| **Renal disease** | Crude | **1 (ref)** | 1.38 (1.08 - 1.75) | 1.69 (1.37 - 2.10) | 2.49 (2.38 - 2.62) | 2.82 (2.37 - 3.35) | 3.21 (2.80 - 3.67) |
|  | Adjusted | **1 (ref)** | 1.33 (1.04 - 1.69) | 1.22 (0.98 - 1.51) | 1.36 (1.29 - 1.43) | 1.47 (1.23 - 1.75) | 1.35 (1.17 - 1.55) |
| **Respiratory disease** | Crude | **1 (ref)** | 1.31 (1.09 - 1.56) | 1.59 (1.35 - 1.87) | 1.32 (1.27 - 1.37) | 1.77 (1.52 - 2.07) | 2.10 (1.86 - 2.36) |
|  | Adjusted | **1 (ref)** | 1.25 (1.05 - 1.50) | 1.31 (1.12 - 1.54) | 1.02 (0.97 - 1.06) | 1.31 (1.12 - 1.53) | 1.39 (1.24 - 1.57) |
| **All-cause mortality** | Crude | **1 (ref)** | 1.13 (0.89 - 1.42) | 1.89 (1.57 - 2.26) | 1.78 (1.70 - 1.86) | 2.00 (1.67 - 2.39) | 2.97 (2.62 - 3.36) |
|  | Adjusted | **1 (ref)** | 1.02 (0.81 - 1.29) | 1.09 (0.90 - 1.30) | 1.01 (0.96 - 1.06) | 0.97 (0.81 - 1.17) | 0.89 (0.78 - 1.01) |

**Key:** body mass index (BMI), confidence interval (CI), cardiovascular disease (CVD), hazard ratio (HR), periodontitis (PD, reference value (ref).
***** Adjusted by age, sex, BMI, ethnicity, average total household income, C-reactive protein level, history of smoking, time since hypertension diagnosis, death and hypertension*PD interaction
